# Pan-cancer analysis of CHRDL1 expression and its mechanistic role in inhibiting EMT via the TGF-β pathway in lung adenocarcinoma

**DOI:** 10.3389/fcell.2025.1557761

**Published:** 2025-03-31

**Authors:** Honghong Dong, Yahui Tian, Shaowei Xin, Yujie Guo, Suxin Jiang, Zitong Wan, Huaiyu Wang, Yong Han

**Affiliations:** ^1^ Department of Thoracic Surgery, Air Force Medical Center, Fourth Military Medical University, Beijing, China; ^2^ Department of Thoracic Surgery, 962 Hospital of the joint Logistics Support Force, Harbin, China; ^3^ Graduate School of China Medical University, Shenyang, China; ^4^ College of Life Sciences, Northwestern University, Xi’an, China

**Keywords:** Chrdl1, lung adenocarcinoma, pan-cancer analysis, Epithelialmesenchymal transition, TGF-β pathway, immune infiltration

## Abstract

**Background:**

The primary objective of this study is to conduct a pan-cancer analysis of CHRDL1 expression, to determine its correlation with patient survival rates, immune cell infiltration, and drug sensitivity. Additionally, the study aimed to further validate the mechanistic role of CHRDL1 in lung adenocarcinoma (LUAD), clarifying its contribution to tumorigenesis and evaluating its potential as a therapeutic target for LUAD.

**Methods:**

We employed bioinformatics strategies to analyze CHRDL1 expression using data from The Cancer Genome Atlas (TCGA) and the Genotype-Tissue Expression Project (GTEx). Survival analysis was executed with GEPIA2, while drug sensitivity to chemotherapeutic agents was evaluated via the CellMiner database. Mutational profiles were examined using cBioPortal, and the immune microenvironment was assessed through the TIMER database. To substantiate our findings, we conducted *in vitro* cellular assays and *in vivo* animal models to validate the mechanistic actions of CHRDL1 in LUAD.

**Results:**

CHRDL1 expression levels showed significant variation across different cancer types, with tumor tissues typically demonstrating lower expression compared to their normal counterparts. In certain cancers, elevated CHRDL1 expression was linked to poorer survival outcomes, whereas in LUAD, it was associated with improved survival. Furthermore, CHRDL1 expression correlated with the IC50 values of multiple chemotherapeutic drugs and played a role in modulating the immune microenvironment. We discovered that CHRDL1 inhibits the epithelial-mesenchymal transition (EMT) in LUAD through the TGF-β pathway.

**Conclusion:**

CHRDL1 exerts a complex influence on cancer development and progression, particularly in LUAD, by impacting tumor progression, immune regulation, chemosensitivity, and EMT regulation. This research offers valuable insights into the overarching mechanisms of cancer progression and aids in the discovery of innovative therapeutic strategies for LUAD treatment.

## 1 Introduction

Cancer is a multifaceted and complex disease, with various molecular mechanisms contributing to its initiation, progression, and resistance to treatment (Bhatia and Kumar, 2014). Over the years, significant advancements in genomics and molecular profiling have provided new insights into the genetic and epigenetic alterations that drive cancer (Berger and Mardis, 2018). The concept of pan-cancer analysis (Wang et al., 2024; Yang et al., 2024) — the study of molecular features across a wide range of cancers—has allowed researchers to identify genes and pathways that are common to various tumor types. This approach has revealed key molecules involved in tumorigenesis, immune evasion, metastasis, and therapeutic resistance, some of which could serve as potential biomarkers or therapeutic targets (Guo et al., 2023; Zhang et al., 2021).

One such molecule that has gained attention in recent years is Chordin-Like 1 (CHRDL1), a member of the chordin-like family of proteins (Berglar et al., 2022; Huang et al., 2024), which are implicated in the regulation of key developmental pathways (Yuan et al., 2024), including the TGF-β signaling pathway (Li et al., 2024). CHRDL1, by modulating the activity of bone morphogenetic proteins (BMPs), plays an important role in regulating TGF-β signaling and extracellular matrix remodeling, both of which are essential in the tumor microenvironment (TME) (Cyr-Depauw et al., 2016).

The implications of CHRDL1 expression in cancer progression remain poorly understood, particularly in terms of its influence on patient prognosis, immune infiltration, and therapeutic sensitivity. These gaps in knowledge are especially evident in lung adenocarcinoma (LUAD), a major subtype of non-small cell lung cancer (NSCLC), where CHRDL1’s function has yet to be fully elucidated. LUAD is one of the deadliest cancers worldwide (Sung et al., 2021), and despite improvements in early detection and targeted therapies, the survival rate remains low, with resistance to therapy and metastasis being major clinical challenges (Kuhn et al., 2018). Understanding the molecular mechanisms underlying LUAD progression is crucial for developing new therapeutic strategies, and CHRDL1 may play a pivotal role in this process.

**FIGURE 1:**
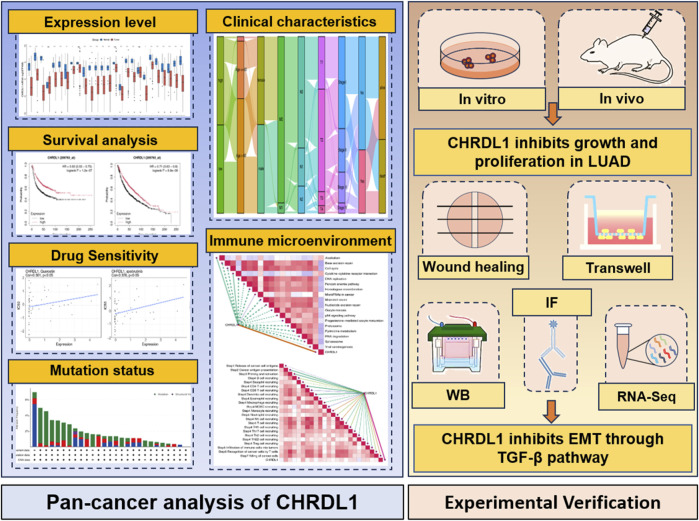
The overview diagram of this study.

This study aims to bridge this knowledge gap by conducting a pan-cancer analysis of CHRDL1 expression across multiple cancer types, with a particular focus on LUAD. We examined the relationship between CHRDL1 expression and patient survival, immune cell infiltration, and drug sensitivity. By leveraging bioinformatics approaches and *in vitro* and *in vivo* experiments, we aim to clarify the role of CHRDL1 in tumorigenesis and its potential as a therapeutic target in LUAD (Figure 1). Our findings could provide critical insights into the broader mechanisms underlying cancer progression and help identify novel strategies for the treatment of LUAD.

## 2 Materials and methods

### 2.1 Data attained and CHRDL1 expression analysis

On 26 September 2024, the transcriptional and clinical data of normal tissues and cancerous tumor tissues (tumor) were acquired from Cancer Genome Atlas (TCGA) database (https://portal.gdc.cancer.gov/), and Genotype-Tissue Expression Project (GTEx) database (http://commonfund.nih.gov/GTEx/), including multi kinds of cancers like bladder urothelial carcinoma (BLCA), LUAD (Supplementary table 1), and gene atlas information of 30 normal tissues. Then expression of CHRDL1 was evaluated. In GTEx dataset, ggradar (v 0.2) package (https://www.rdocumentation.org/packages/ggradar/versions/0.2/topics/ggradar-package) was employed sed to draw a radar chart to display expression of CHRDL1 in 31 normal tissues. In TCGA dataset, wilcoxon test was adopted to contrast differential expression of CHRDL1 ranging from tumor group to normal group (p < 0.05), and ggplot2 (v 3.3.6) package (Gustavsson et al., 2022) was utilized for visualization. The differential expression of CHRDL1 among tumor and normal groups in TCGA and GTEx datasets was simultaneously performed through SangerBox website (http://SangerBox.com/Tool) using Wilcoxon test (p < 0.05). The SangerBox website integrated several important databases such as Gene Expression Omnibus (GEO), TCGA and International Cancer Genome Consortium (ICGC), and contained information on multiple cancer types within these databases.

### 2.2 Survival prognosis analysis of CHRDL1

Survival atlas data of overall survival (OS) and recurrence free survival (RFS) of CHRDL1 in diverse cancer types within TCGA database were attained with aid of online website gene expression profiling interactive analysis (GEPIA 2 (cancer-pku.cn)). Based on two expression thresholds, namely high cut-off value (50%) and low cut-off value (50%), high- and low-expression cohort of CHRDL1 were attained accordingly. Subsequently, log-rank test was adopted to explore distinctions between groups (p < 0.05), and “survival analysis” module of GEPIA2 was utilized to study and visually display special survival plots with log-rank p values. And hazard ratio (HR) value was calculated. When HR was more than 1, it was regarded as a risk factor. When HR was less than 1, it was considered a protective factor. And when HR was equal to 1, it was deemed to be meaningless. Immediately after that, connections between expression level of CHRDL1 and OS as well as disease specific survival (DSS) of patients with different cancers were dissected on SangerBox portal. The forestplot (v 3.1.1) package (Li et al., 2022) was employed to conduct univariate Cox analysis, and meanwhile, HR and 95% confidence interval (CI) were assessed (p < 0.05).

Furthermore, in GEO, European Genome - phenome Archive (EGA) and TCGA databases, associations between expression of CHRDL1 and prognosis of patients with ovarian cancer (OV), LUAD, stomach adenocarcinoma (STAD) and liver hepatocellular carcinoma (LIHC) (Wei et al., 2022) were further assessed. The survival (v 3.5–3) package (Lei et al., 2023) was employed for plotting operations on Kaplan-Meier (K-M) survival curve plotting website (https://kmplot.com/analysis/). In “mRNA gene chip” and “mRNA RNA-seq” modules, K-M survival curves of patients with OV, LUAD, STAD and LIHC were generated (p < 0.05), and relationships between expression of CHRDL1 and OS, progression free survival (PFS), first progression (FP), post progression survival (PPS), and RFS in prognosis of cancer patients were deeply evaluated.

### 2.3 Chemotherapeutic drug sensitivity analysis

The RNA expression data and chemotherapeutic drug sensitivity data of cancer cell lines were attained from CellMiner database (https://discover.nci.nih.gov/CellMiner/home.do). Then, chemotherapeutic drugs that had undergone clinical trials and been authorized by food and drug administration (FDA) were selected for analysis. Subsequently, half-maximal inhibitory concentrations (IC_50_) values of drugs were gained. The psych (v 2.2.9) package (Author anonymous, 2023) was utilized to conduct Spearman connection analysis on expression data of CHRDL1 and IC_50_ values, as well as connection analysis between CHRDL1 and IC_50_ values of drugs for LUAD (|correlation (cor) | > 0.3, p < 0.05).

### 2.4 Analysis of the mutation characteristics of CHRDL1

In TCGA dataset, cBioPortal tool (http://www.cbioportal.org/) was employed to study mutation characteristics of CHRDL1 in different cancers. The lollipop plot was employed to display all mutation sites of CHRDL1. The log-rank test in survival (v 3.5–3) package was utilized to conduct K-M analysis. The survival differences in disease - free survival (DFS), DSS, PFS, and OS were respectively compared between pan-cancer patients with CHRDL1 mutations and those without mutations, as well as between LUAD patients with CHRDL1 mutations and those without mutations (p < 0.05).

### 2.5 Immune microenvironment analysis

To explore infiltration levels of immune cells in 32 types of cancers, association analysis between immune cells and CHRDL1 was carried out by “GENE” module of Tumor Immune Estimation Resource (TIMER) database (http://timer.cistrome.org/) (|cor| > 0.3, p < 0.05). Subsequently, association between CHRDL1 and immune cells in LUAD was calculated by seven algorithms (xCell immune infiltration analysis tool (XCELL), TIMER, Quantification of cell types from bulk tissue gene expression data using signature matrices (QUANTISEQ), Microenvironment Cell Populations - Counter (MCPCOUNTER), Cell - type Identification By Estimating Relative Subsets Of RNA Transcripts (IBERSORT), IBERSORT-ABS, Estimating Proportion of Immune and Cancer cells (EPIC)) (|cor| > 0.3, p < 0.05). Similarly, Spearman correlation analysis was conducted between CHRDL1 and 122 immune modulators (Feng et al., 2022) (major histocompatibility complex (MHC), chemokines, receptors, and immunostimulants) (Supplementary Table S2), immune checkpoints (Hu et al., 2021; Yu et al., 2021), LUAD immune cycle activity, and immune therapy prediction pathway enrichment scores via psych (v 2.2.9) package (|cor| > 0.3, p < 0.05). The pheatmap (v 1.0.12) package (Gu and Hubschmann, 2022) was utilized to draw heatmap to display the results.

### 2.6 The clinical characteristics of CHRDL1 in LUAD

In TCGA-LUAD dataset, ggalluvial (v 0.12.5) package (Brunson, 2020) was utilized to analyze expression of CHRDL1 in subgroups with different clinical characteristics (age (≤60, >60), gender (male, female), M stage (M0, M1), N stage (N0, N1, N2), T stage (T1, T2, T3, T4), stage (Ⅰ, Ⅱ, Ⅲ, Ⅳ), and smoking (smoke) (no, yes), status (alive, death)), and an alluvial plot was drawn for visualization. Subsequently, in TCGA-LUAD and GEO datasets, Wilcoxon test was performed to analyze expression of CHRDL1 among subgroups with LUAD clinical characteristics (p < 0.05). The clinical characteristics in TCGA-LUAD dataset including (age, gender, T stage, N stage, M stage, stage, smoking). The GSE31210 dataset was downloaded from GEO dataset. In GSE31210, clinical information comprised age, gender, stage, and smoking.

### 2.7 Correlation between CHRDL1 and treatment feature enrichment scores as well as drug target genes in LUAD

The drug target genes related to LUAD were gained from Therapeutic Target Database (TTD) (idrblab.net). The psych (v 2.2.9) package was used to perform Spearman correlation analysis between CHRDL1 and drug target genes as well as treatment feature enrichment scores (|cor| > 0.3, p < 0.05).

### 2.8 Statistical analysis

In this study, bioinformatics analysis was conducted via R (v 4.2.2) language. A p value was under 0.05 was taken as statistically meaningful. The Wilcoxon test was employed to perform analysis of distinctions between groups. Experimental Statistics were performed using GraphPad Prism 8.0. All experimental data were analyzed by Student’s t-test (two-tailed), and P < 0.05 was considered statistically significant. All error bars represent SEM.

### 2.9 Plasmid

The pCDH-EF1a-human-CHRDL1-3×Flag plasmid was purchased from Tsingke. psPAX2 and pMD2. G were purchased from Addgene. The shCHRDL1#1 (TRCN0000149739) and shCHRDL1#2 (TRCN0000371790) (Berglar et al., 2022) were purchased from Tsingke.

### 2.10 Cell lines and cell culture

Human LUAD cell lines (A549, 838, Hop62 and PC-9) was kindly provided by laboratory colleagues. All cells were cultured in RPMI-1640 (Gibco) supplemented with 10% fetal bovine serum (FBS) (Procell) with standard conditions at 37°C, 5% CO2, and a humidified atmosphere.

### 2.11 Human tissues

The human lung cancer samples used in this study were obtained from the biobank of Sunshine Union Hospital. We randomly selected paraffin samples from 15 LUAD patients to construct tissue microarrays. The sampling locations for both tumor and normal tissues were determined by two pathology experts, ensuring that the normal tissue was located more than 3 cm away from the tumor margin. The study protocol was approved by the Human Ethics Committee of Weifang Medical University, and informed consent was obtained from all patients at the time of sample collection. All work was performed following the approved protocol.

### 2.12 RNA extraction and RT-qPCR

Total RNA was extracted with Trizol reagent (Invitrogen), data shown are the relative abundance of the indicated mRNA normalized to that of GAPDH. Quantitative reverse transcription PCR was performed using the following gene specific primers

(5′-3′):
CHRDL1-F: CCTGGAACCTTATGGGTTGGT
CHRDL1-R: AACATTTGGACATCTGACTCGG
GAPDH-F: CAAGTATGATGACATCAAGAAGGTGG
GAPDH-R: GGAAGAGTGGGAGTTGCTGTTG

### 2.13 Cell proliferation assay

1 × 10^3^ cells were seeded in 96-well plates in RPMI medium 1,640 containing 10% FBS for 1–6 days. Proliferation activity was measured using Cell Counting Kit-8 (CCK8, Dojindo Molecular Technologies) following the manufacturer’s protocol.

### 2.14 Colony-forming assay

0.5 × 10^3^ cells were seeded in 6-well plates in RPMI medium 1,640 containing 10% FBS medium. After 2 weeks, the cells were washed with 1 × PBS, fixed with formaldehyde for 10 min, and stained with 0.05% crystal violet for 30 min. Colonies were counted by ImageJ software.

### 2.15 Western blot

Using RIPA buffer (Beyotime) containing phenylmethylsulfonyl fluoride (PMSF) and NaF (Beyotime) (RIPA: PMSF: NaF = 100:1:1) to lyse cells and extract total protein. Measure protein concentration using BCA protein assay kit (NCM). Then, 10% polyacrylamide gel (sodium dodecyl sulfate polyacrylamide gel electrophoresis) was used for electrophoresis. Transfer the protein onto a PVDF membrane and seal with 5% skim milk for 1 h. The membranes were placed in primary antibodies for overnight, and the washed by TBST 3 times for 10 min each time. Incubate the secondary antibody at room temperature for 1 h, wash the membrane with TBST three times for 10 min each time. Finally, the bands were imaged with a Bio-Rad gel imaging system (Bio-Rad) and were analyzed with ImageJ software (version 1.54e), normalized to GAPDH.

### 2.16 Immunohistochemistry (IHC)

The sections were immersed in xylene twice for 10 min each to remove the paraffin. Then, they were sequentially immersed in 100%, 95%, 80%, and 70% ethanol for 5 min each to complete hydration. Finally, the sections were incubated in PBS for 5 min to wash off the ethanol. The sections were placed in antigen retrieval solution and heated in a water bath at 95 °C for 15 min. Afterward, the sections were washed with PBS three times, each for 5 min. To block endogenous peroxidase activity, the sections were incubated in 3% H_2_O_2_ solution for 25 min, followed by three PBS washes of 5 min each. Next, 3% BSA was evenly applied to cover the tissue, and the sections were blocked at room temperature for 30 min. The blocking solution was gently discarded. Slice with primary antibody CHRDL1 (162,463; 1:300), E-cadherin (GB12083, Servicebio, 1:5,000), N-Cadherin (GB12135, Servicebio, 1:1,500), Vimentin (GB121308, Servicebio, 1:1,000), Claudin-1 (GB152543, Servicebio, 1:1,000), and Snail (GB11260, Servicebio, 1:1,000) were incubated overnight at 4°C. The next day, after washing with PBS, the diluted secondary antibody solution was applied, and the sections were incubated at room temperature for 60 min. Following PBS washes, DAB chromogenic solution was applied, and the color development time was controlled under the microscope, with positive staining appearing brownish-yellow. PBS was used to wash the sections to stop the reaction. The nuclei were counterstained with hematoxylin, followed by PBS washing. Finally, the sections were dehydrated, mounted, and observed under a microscope after drying. Using the “IHC Profiler” plugin in ImageJ, we selected the appropriate staining mode to obtain the intensity scores of the positive staining areas. The data were then imported into GraphPad Prism 8.0 for further analysis.

### 2.17 *In vivo* tumorigenesis

Ten 6-week-old nude mice were randomly divided into two groups: the experimental group and the control group, with 5 mice in each group. Subcutaneous injections of 5 × 10^6^ corresponding lung adenocarcinoma cells were administered in the right axillary region of each mouse. The cells are suspended in 100 μL of PBS containing 50% Matrigel basement membrane matrix. Begin measuring the size of the tumor on day 7 (the sixth day post-inoculation), and measure every 2 days thereafter. Use calipers to measure the longest and shortest dimensions of the implanted tumor, which correspond to the length (a) and width (b), respectively. Calculate the tumor volume using formula V = 1/2 × a × (b)^2^. The tumor volume is expressed in cubic millimeters (mm^3^). Perform small animal *in vivo* imaging when the tumor diameter reaches approximately 10 mm. The collected tissue was immediately fixed in 4% paraformaldehyde for 24 h. The tissue was then trimmed to an appropriate shape and sequentially processed through dehydration using ethanol solutions of different concentrations. The tissue was subsequently cleared with xylene and then embedded in paraffin. Finally, the tissue was embedded to form a complete paraffin block.

### 2.18 Transwell

Seed 5 × 10^4^ cells onto the upper chamber of a Transwell insert, either with or without Matrigel matrix, while the lower chamber is filled with 600 μL of complete growth medium containing FBS. Culture the cells in the incubator for 24–48 h. Fix the upper chamber with paraformaldehyde and stain with crystal violet. Observe and photograph the migration and invasion of cells under a microscope, and process the experimental results using ImageJ.

### 2.19 Wound healing

Mark horizontal lines on the back of a 6-well plate with intervals of 0.5–1 cm for ease of observation and documentation. Seed LUAD cells onto the 6-well plate and culture them overnight. Allow the cells to grow until they reach a confluence of over 90%. Utilize a 200 μL pipette tip to create uniform-width scratches in a vertical direction across the cell monolayer. Rinse each well with Phosphate-Buffered Saline (PBS) three times to remove any detached cells. Add serum-free medium to each well to facilitate cell migration without the influence of serum factors. Observe and photograph the wells at 0 h, 12 h, and 24 h to document the progression of cell migration into the scratch area. Process the experimental results using ImageJ software to analyze cell migration patterns and closure of the scratch.

### 2.20 Immune fluorescence

Cultured cells were fixed in 4% paraformaldehyde (PFA) for 15 min at room temperature (RT), followed by washing with PBS. To permeabilize the cell membrane, the samples were incubated with 0.2% Triton X-100 in PBS for 15 min at RT. Non-specific binding was blocked by incubating the samples with 3% BSA in PBS for 1 h at RT. The glass slides were then incubated with the primary antibody overnight at 4°C, followed by washing with PBS. Afterward, the glass slides were incubated with fluorophore-conjugated secondary antibody for 1 h at RT in the dark. Nuclear staining was performed using DAPI (1:1,000) for 5 min at RT. Finally, the samples were mounted with anti-fade mounting medium and visualized using a fluorescence microscope (Zeiss LSM 800). Fluorescence intensity was quantified using ImageJ software.

## 3 Results

### 3.1 CHRDL1 expression variation across cancers and tissues

**FIGURE 2:**
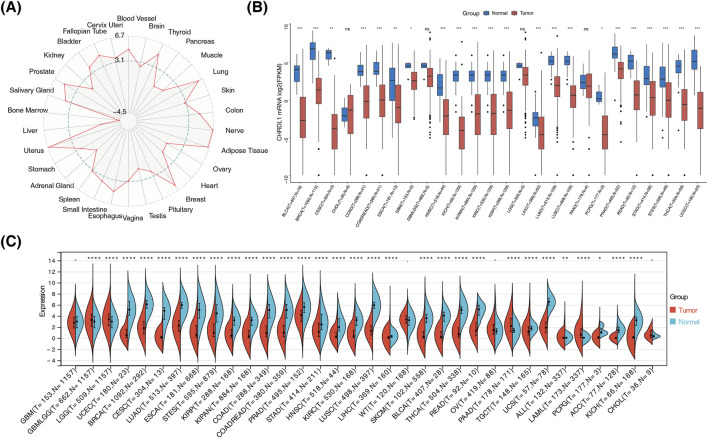
CHRDL1 expression variation across cancers and tissues. **(A)** Expression of CHRDL1 in GTEX database. **(B)** Expression of CHRDL1 in TCGA database. **(C)** Expression of CHRDL1 in combination of GTEX and TCGA databases. (**** p < 0.0001, *** p < 0.001, ** p < 0.01, *p < 0.05, ^ns^p > 0.05).

The GTEx dataset revealed low CHRDL1 expression in bone marrow and liver, with peak levels in nerve, uterus, and adipose tissues among 30 analyzed tissues (Figure 2A). In TCGA dataset, significant differences in CHRDL1 expression were identified among 22 cancer types, with tumor tissues exhibiting reduced expression compared to normal tissues (p < 0.05), notably in bladder urothelial carcinoma (BLCA), breast invasive carcinoma (BRCA), cervical squamous cell carcinoma (CESC), and colon adenocarcinoma (COAD) (Figure 2B). Comparative analysis of both datasets highlighted significant expression disparities between most cancer types and their corresponding normal tissues (p < 0.05) (Figure 2C). These findings underscore the potential of CHRDL1 as a biomarker and therapeutic target, warranting further investigation into its mechanistic role in disease pathology.

### 3.2 Different impacts of CHRDL1 expression level on multiple key survival indicators of different cancers

**FIGURE 3:**
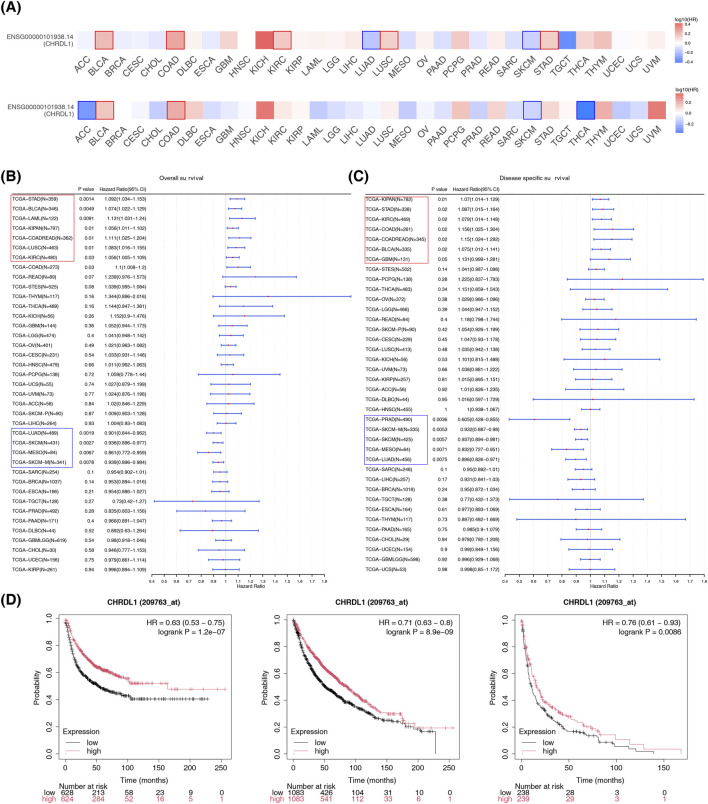
Different impacts of CHRDL1 expression level on multiple key survival indicators of different cancers. **(A)** OS and RFS with high and low expression of CHRDL1 in various cancers. Cox regression analysis of CHRDL1 expression and OS **(B)** and DSS **(C)** in various cancers. **(D)** The FP, OS and PPS of LUAD.

In BLCA (HR = 1.6, p = 0.0028), COAD (HR = 1.8, p = 0.015), kidney renal clear cell carcinoma (KIRC) (HR = 1.5, p = 0.015), lung squamous cell carcinoma (LUSC) (HR = 1.4, p = 0.022), and STAD (HR = 1.4, p = 0.036), OS was lower in high-expression group of CHRDL1, while in LUAD (HR = 0.66, p = 0.0062) and skin cutaneous melanoma (SKCM) (HR = 0.71, p = 0.012), OS was lower in low-expression group of CHRDL1. In BLCA (HR = 1.4, p = 0.038), and COAD (HR = 1.7, p = 0.039), RFS was lower in high-expression group of CHRDL1, and in adrenocortical carcinoma (ACC) (HR = 0.49, p = 0.041), SKCM (HR = 0.76, p = 0.025), and thyroid carcinoma (THCA) (HR = 0.4, p = 0.0028), RFS was lower in low-expression group of CHRDL1(Figure 3A; Supplementary Figure S1A, B). The results of univariate Cox regression analysis demonstrated that OS of 12 types of cancers was substantially associated with expression level of CHRDL1 (p < 0.05). Among them, expression of CHRDL1 was a risk factor for 8 types of cancers, including STAD, BLCA, acute myeloid leukemia (LAML), kidney pancreatic cancer (KIPAN), COAD-rectum adenocarcinoma (COAD-READ), lung squamous cell carcinoma (LUSC), KIRC, and COAD (HR > 1, p < 0.05), while it was a protective factor for 4 types of cancers, namely LUAD, SKCM, mesothelioma (MESO), and SKCM-metastatic (SKCM-M) (HR < 1, p < 0.05) (Figure 3B). The DSS of 12 types of cancers was also dramatically related to expression level of CHRDL1. Specifically, expression of CHRDL1 was a risk factor for 7 types of cancers, including KIPAN, STAD, KIRC, COAD, COADREAD, and BLCA (HR > 1, p < 0.05), and it was a protective factor for 5 types of cancers, such as prostate adenocarcinoma (PRAD), SKCM-M, SKCM, MESO, and LUAD (HR < 1, p < 0.05) (Figure 3C). The K-M survival curve indicates that in patients with LUAD, those with high expression of CHRDL1 have significantly better FP, OS, and PPS (Figure 3D). Additionally, in patients with OV, those with high expression of CHRDL1 have significantly lower OS and PFS compared to those with low expression of CHRDL1 (HR > 1, p < 0.05), and patients with low expression of CHRDL1 have significantly lower PPS compared to those with high expression of CHRDL1 (HR = 0.83, 95% CI 0.69–1, p = 0.046) (Supplementary Figure S1C).

### 3.3 Correlation of CHRDL1 expression with chemotherapeutic drug sensitivity

**FIGURE 4:**
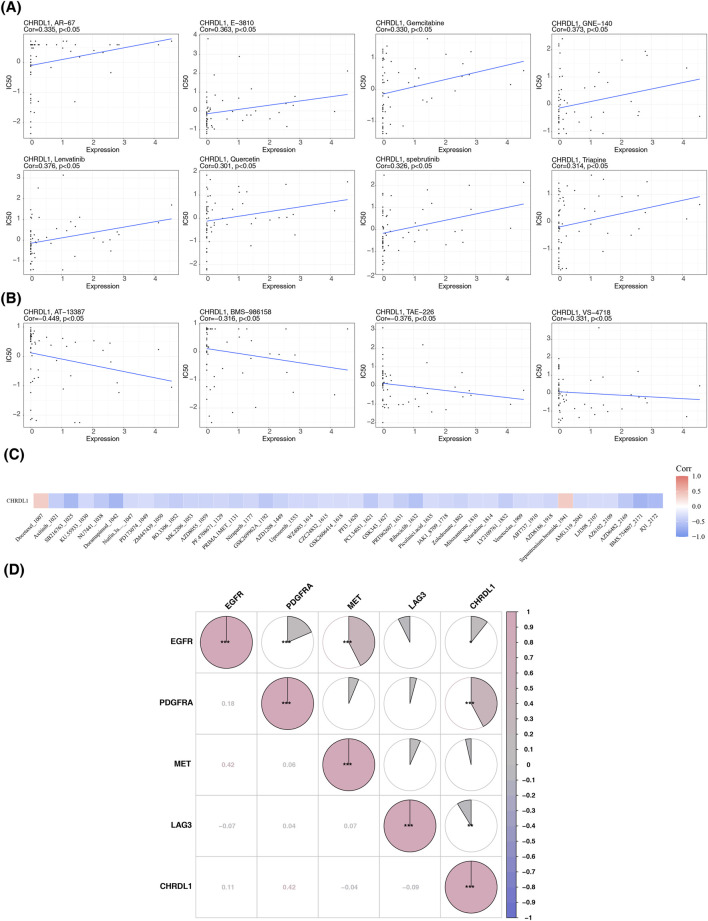
Correlation of CHRDL1 expression with chemotherapeutic drug sensitivity. **(A)** Eight drugs with a positive correlation between IC50 values and CHRDL1 expression. **(B)** Four drugs with a negative correlation between IC50 values and CHRDL1 expression. **(C)** Drugs correlated with CHRDL1 expression in LUAD. **(D)** Correlation between drug-related target genes and CHRDL1 in LUAD.

CHRDL1 expression significantly correlates with the IC_50_ values of 12 chemotherapeutic agents. Positive correlations were observed with 8 drugs, including AR-67, E−3810, gemcitabine, GNE-140, lenvatinib, quercetin, spebrutinib, and troapine (correlation coefficient >0.3, p < 0.05). Conversely, negative correlations were noted with 4 drugs: AT-13387, BMS-986158, TAE-226, and VS-4718 (correlation coefficient < −0.3, p < 0.05) (Figures 4A, B). In LUAD, CHRDL1 expression was associated with 42 drugs, with positive correlations for docetaxel 1,007 and sepantronium bromide 1941 (correlation coefficient >0.3), and negative correlations for 40 other drugs (Figure 4C). Additionally, CHRDL1 showed a substantial positive correlation with PDGFRA, a drug target gene in LUAD (correlation coefficient = 0.42, p < 0.05) (Figure 4D).

### 3.4 The mutation status of CHRDL1 in pan-cancer and LUAD

**FIGURE 5:**
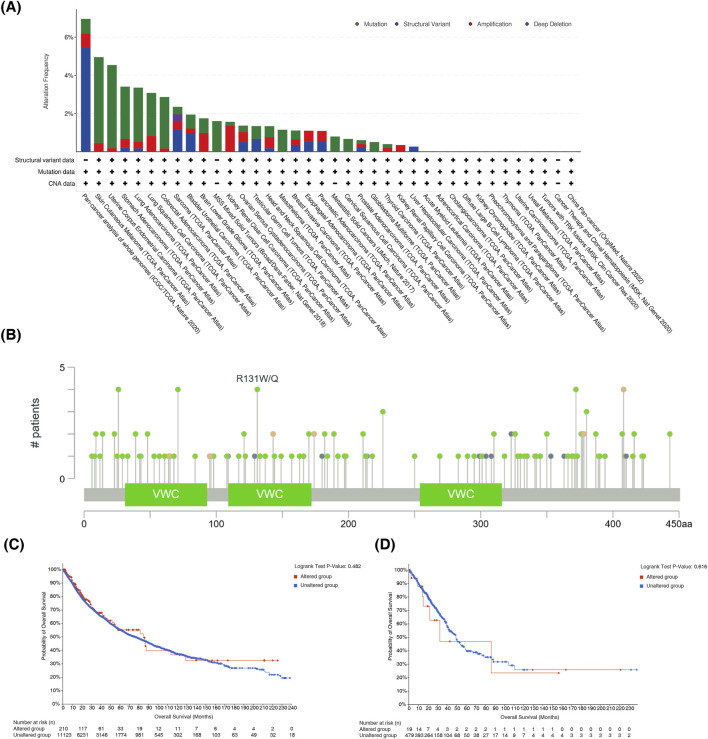
The mutation status of CHRDL1 in pan-cancer and LUAD. **(A)** Prevalence of different mutation types of CHRDL1 in Pan-Cancer. **(B)** Visualization of CHRDL1 mutational hotspots in Pan-Cancer. Association between CHRDL1 mutation status and patient outcomes in Pan-Cancer **(C)** and LUAD **(D)**.

CHRDL1 was mutated in multiple cancers. The greatest mutation rate was observed in SKCM, succeeded by uterine corpus endometrial carcinoma (UCEC), STAD and LUAD and most common mutation type was missense mutation (Figure 5A). In general, in pan-cancer, most frequent mutation type of CHRDL1 was missense mutation (Figure 5B). And in pan-cancer patients, there was no substantial variance in DFS, DSS, OS and PFS between patients with CHRDL1 mutation and those without (p > 0.05) (Figure 5C; Supplementary Figure S2A). In LUAD, there was also no significant difference in DFS, DSS, OS and PFS between patients with CHRDL1 mutation and those without (p > 0.05) (Figure 5D; Supplementary Figure S2B).

### 3.5 The impact of CHRDL1 on the cancer immune microenvironment

**FIGURE 6:**
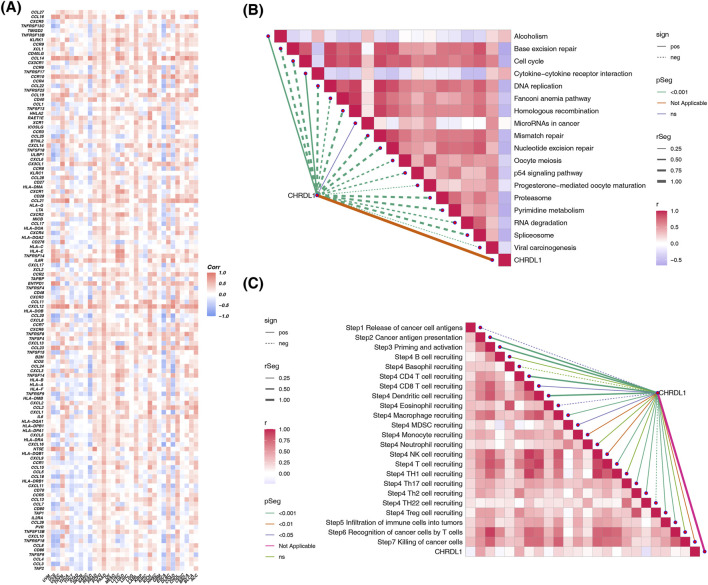
The impact of CHRDL1 on the cancer immune microenvironment. **(A)** Correlation of CHRDL1 expression with immune modulators in pan-cancer. Association of CHRDL1 with immunotherapy predictive pathways **(B)** and immune cycle activity scores **(C)** in LUAD.

In 21 type of cancers, including pancreatic adenocarcinoma (PAAD), COAD, STAD, READ, LUAD, LUSC, CHRDL1 showed strong positive correlations with hematopoietic. stem.cell_Xcell (cor >0.3, p < 0.05), with PAAD exhibiting the highest (cor = 0.77, p < 2.2*10^−16^) (Supplementary Figure S3A). In UCEC, PRAD, KICH and testicular germ cell tumor (TGCT), CHRDL1 was significantly negatively correlated with class. switched.memory.B.cell_Xcell (|cor| > 0.3, p < 0.05), with TGCT showing the highest negative correlation (cor = −0.59, p < 7.5e-16) (Supplementary Figure S3B). CHRDL1 exhibits different correlations with immune modulators in various tumors. In LUAD, CHRDL1 is positively correlated with CXCR1 (cor = 0.759), CCL16 (cor = 0.734), and CCL21 (cor = 0.477). In COAD, CHRDL1 is negatively correlated with TAP1 (cor = −0.589), CXCL11 (cor = −0.604), and CXCL16 (cor = −0.647) (Figure 6A; Supplementary Table S3). In LUAD, CHRDL1 is significantly positively correlated with 16 immune checkpoint genes, including CD40LG (cor = 0.59), CD44 (cor = 0.44), and LAIR1 (cor = 0.44) (all p < 0.05) (Supplementary Figure S3C). CHRDL1 is negatively correlated with most immune therapy predictive pathways, such as base excision repair, cell cycle, mismatch repair, etc. (cor >0.3, p < 0.05) (Figure 6B). In the immune cycle activity scores of LUAD, CHRDL1 is significantly positively correlated with Step2 cancer antigen presentation (cor = 0.472, p < 0.05) and Step4 CD4 T cell recruitment (cor = 0.435, p < 0.05) (Figure 6C; Supplementary Table S4).

### 3.6 CHRDL1 expression across diverse clinical characteristics of LUAD

**FIGURE 7:**
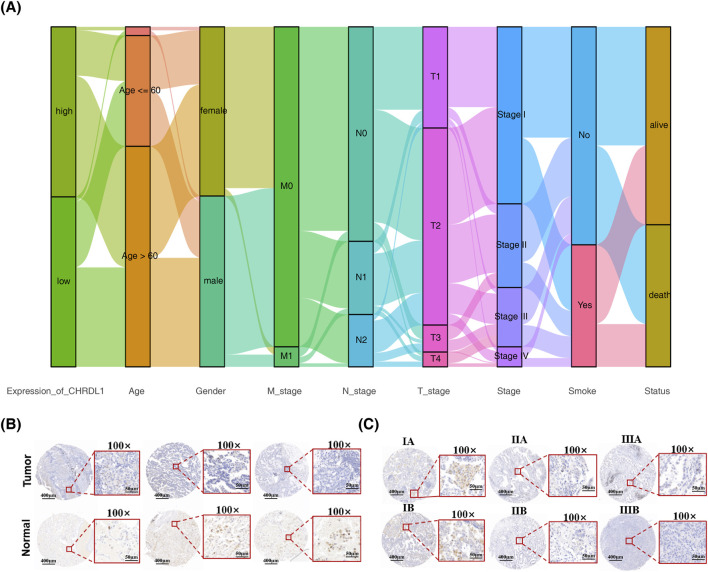
CHRDL1 expression across diverse clinical characteristics of LUAD. **(A)** Clinical feature differences based on CHRDL1 expression levels in LUAD patients. **(B)** Immunohistochemical staining showing CHRDL1expression in tumor and normal tissues of LUAD patients. Scales represent respectively distances of 400 µm and 50 µm. **(C)** Immunohistochemical staining showing chrdl1 expression in LUAD patients at different tumor stages. Scales represent respectively distances of 400 µm and 50 µm.

Upon constructing a stratification impact plot utilizing gene expression data from patients with LUAD, it was observed that individuals with elevated CHRDL1 expression predominantly comprised females and those with early-stage T, N, and M classifications (Figure 7A). Subsequent analysis within the TCGA-LUAD cohort confirmed a negative correlation between CHRDL1 expression levels and the progression of T, N, and M tumor stages. Notably, increased expression was associated with patients above the age of 60, non-smokers, and the female demographic (Supplementary Figure S4A). Consistent with these findings, the GSE31210 dataset corroborated these observations (Supplementary Figure S4B). Immunohistochemical assays were performed on tissue specimens from patients with LUAD, revealing significantly higher CHRDL1 expression in normal tissues compared to adenocarcinoma tissues (Figure 7B). Furthermore, CHRDL1 expression levels decreased with advancing tumor stages of LUAD (Figure 7C), aligning with prior bioinformatics analyses and substantiating the correlation between CHRDL1 expression and clinical pathology.

### 3.7 Elevated CHRDL1 expression suppresses the growth and proliferation of LUAD cells

**FIGURE 8:**
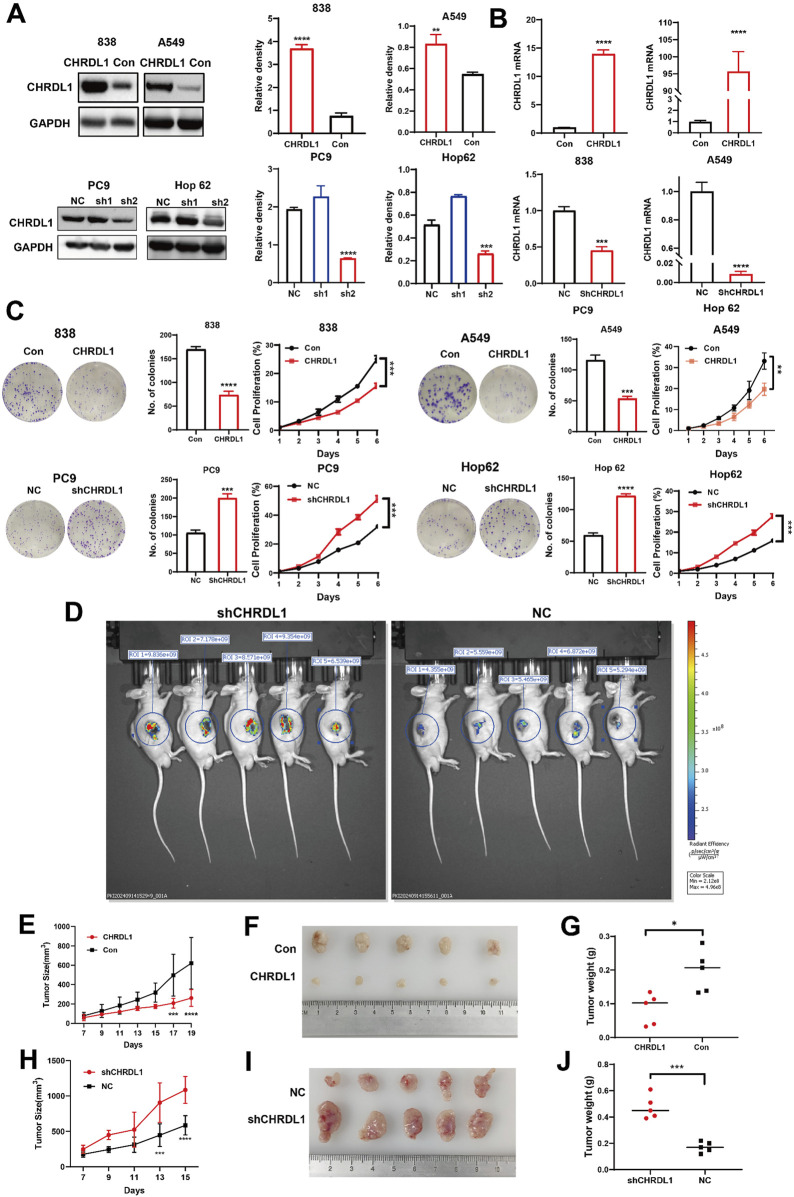
CHRDL1 inhibits the growth and proliferation of LUAD cells both in vivo and *in vitro*. WB assay **(A)** and RT-PCR assay **(B)** confirming successful construction of LUAD cell lines with high (838/A549) and low (PC9/Hop62) CHRDL1 expression. **(C)** Clone formation assay and CCK8 assay conducted on LUAD cell lines with high (838/A549) and low (PC9/Hop62) CHRDL1 expression. **(D)** *In vivo* imaging of tumors in NC and shCHRDL1 groups in nude mice. **(E)** Growth curves of subcutaneous xenografts in nude mice for CHRDL1 high-expression (838) LUAD cell lines. **(F)** Photographs of tumors derived of high-expression LUAD cell lines. **(G)** Tumor weights from high-expression LUAD cell lines. **(H)** Growth curves of subcutaneous xenografts in nude mice for CHRDL1 low-expression LUAD cell lines. **(I)** Photographs of tumors derived of low-expression LUAD cell lines. **(J)** Tumor weights of low-expression LUAD cell lines.

We established LUAD cell lines with elevated (838/A549) and reduced (PC9/Hop62) CHRDL1 expression, confirmed by Western blot and RT-PCR. Western blot analysis indicated that sh2 was more effective in knockdown of CHRDL1, henceforth denoted as shCHRDL1 (Figures 8A, B). Subsequent colony formation and CCK8 assays on these cells revealed that CHRDL1 overexpression reduced clonal formation and proliferation, whereas CHRDL1 knockdown increased clonal formation both, promoting cell proliferation (Figure 8C).

### 3.8 CHRDL1 inhibits the growth and proliferation of LUAD in vivo

To further investigate the effects of CHRDL1 on the growth and proliferation of LUAD cells *in vivo*, we subcutaneously injected LUAD cells with either high (838) or low (PC9) expression levels of CHRDL1 into nude mice and monitored the tumor formation. Small animal imaging was performed 14 days post-tumor inoculation, revealing significantly higher fluorescence intensity in the shCHRDL1 group, indicating that downregulation of CHRDL1 promotes tumor growth (Figure 8D). Tumors were harvested when they reached maturity. The results demonstrated that, compared to the control group, the group with high CHRDL1 expression exhibited significantly smaller and lighter subcutaneous tumors (Figures 8E–G). Conversely, downregulation of CHRDL1 led to a marked increase in both the volume and weight of subcutaneous tumors (Figures 8H–J). These findings suggest that CHRDL1 can inhibit the growth and proliferation of LUAD cells *in vivo*.

### 3.9 CHRDL1 inhibits the EMT pathway in LUAD cells

**FIGURE 9:**
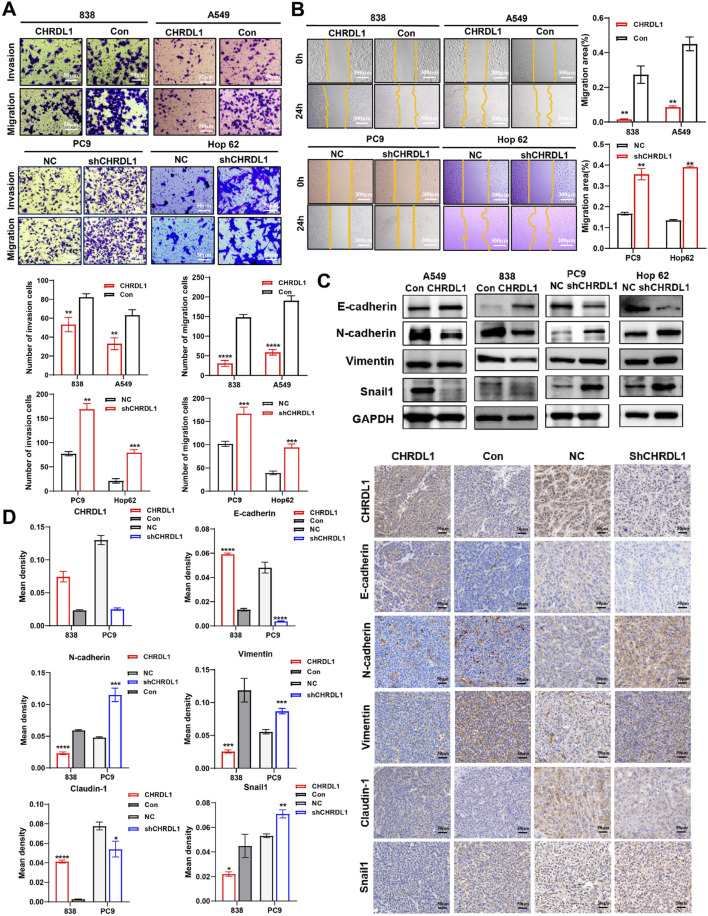
CHRDL1 inhibits the EMT pathway in LUAD cells.Results of transwell **(A)** and wound healing **(B)** assay of LUAD cells with high and low CHRDL1 expression. Scales represent respectively distances of 50 and 300 µm. **(C)** Detection of EMT-related molecule expression levels. **(D)** Immunohistochemical staining results of EMT-related molecules in xenograft tumors. Scale bar represents a distance of 50 µm.

Knockdown of CHRDL1 induces morphological changes in LUAD cells, which appear more elongated compared to the negative control (NC) group (Supplementary Figure S5). Corroborating previous bioinformatics analysis, which showed a negative correlation between CHRDL1 expression and tumor staging, we hypothesized that CHRDL1 might influence the invasive and metastatic capabilities of LUAD cells. Transwell assays with cells overexpressing and underexpressing CHRDL1 revealed significantly impaired invasion and migration in the overexpression group, while knockdown of CHRDL1 enhanced these capabilities (Figure 9A). Wound healing assays confirmed that CHRDL1 overexpression inhibits tumor cell migration, and knockdown increases it (Figure 9B). These results suggest that CHRDL1 suppresses the invasive and metastatic potential of LUAD cells. To assess the impact of CHRDL1 on epithelial-mesenchymal transition (EMT), we detected EMT-related markers and found that CHRDL1 overexpression elevated epithelial markers (E-cadherin) and decreased mesenchymal markers (N-cadherin, Vimentin, Snail1). Conversely, in the underexpression group, E-cadherin was reduced, while N-cadherin, Vimentin, and Snail1 were increased, indicating that CHRDL1 inhibits the EMT pathway (Figure 9C). Furthermore, immunohistochemical staining of subcutaneous tumors in nude mice (Figure 9D) showed increased expression of E-cadherin and claudin-1, and decreased expression of N-cadherin, Vimentin, and Snail1 in the overexpression group, with the opposite observed in the underexpression group. Collectively, these findings indicate that CHRDL1 inhibits the EMT pathway and reduces the expression of EMT-related molecules.

### 3.10 CHRDL1 regulates EMT via the TGF-β pathway

**FIGURE 10:**
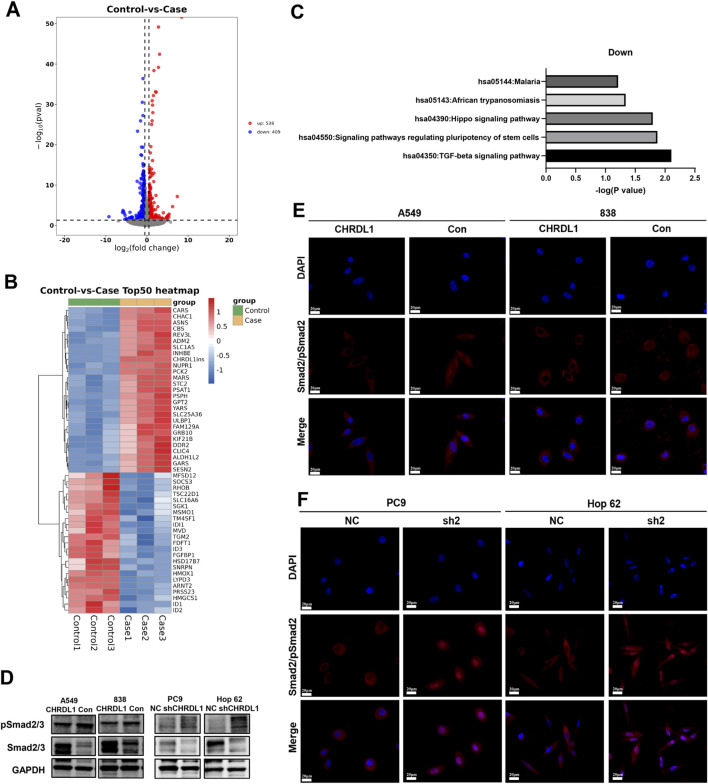
CHRDL1 Regulates EMT via the TGF-β Pathway. **(A)** Volcano plot of differential gene expression in CHRDL1-overexpressing cells (838). **(B)** Heatmap of gene expression in CHRDL1-overexpressing cells. **(C)** KEGG analysis of CHRDL1 overexpression. **(D)** Detection of TGF-β pathway-related molecules expression. **(E–F)** Immunofluorescence shows the effect of CHRDL1 on pSmad2 nuclear translocation. Scale bar represents a distance of 50 µm.

Transcriptome sequencing was performed on cells with high expression of CHRDL1, and differential gene analysis identified genes that are positively and negatively correlated with CHRDL1 expression (Figures 10A, B). Subsequent KEGG analysis revealed that the TGF-β pathway was significantly inhibited following high expression of CHRDL1 (Figure 10C). WB experiments found that the expression of pSmad2/3 was markedly reduced in cells with high CHRDL1 expression, whereas knockdown of CHRDL1 led to a significant increase in pSmad2/3 expression (Figure 10D). Additionally, we conducted immunofluorescence experiments and found that knockdown of CHRDL1 significantly promotes the nuclear translocation of PSMAD2 (Figures 10E, F). These results demonstrate that CHRDL1 regulates the EMT pathway through the TGF-β/Smad pathway.

## 4 Discussion

Our study extensively explored the expression patterns and prognostic value of CHRDL1 in pan-cancer. Our analysis revealed that CHRDL1 is lowly expressed in most tumors, with its expression level closely associated with patient prognosis. Specifically, low CHRDL1 expression is correlated with poor survival outcomes in melanoma and LUAD, while the opposite is true in BLCA and COAD. Previous studies (Deng et al., 2022; 2021; Yuan et al., 2024) have identified CHRDL1 as a prognostic marker for LUAD, with lower expression observed in low-risk gastric cancer patients (Hu et al., 2018). In oral squamous carcinoma, high mRNA levels of CHRDL1 suggest poor prognosis (Han et al., 2023). Additionally, CHRDL1 shows prognostic value in breast cancer (Wang et al., 2020), thyroid cancer (Pan et al., 2021; Ren et al., 2021), and clear cell renal carcinoma (Wu et al., 2023). These findings indicated that CHRDL1 exhibits strong prognostic relevance in cancers and may play a role in regulating various tumor biological processes. Mutational analysis also suggested that clinical detection of CHRDL1 mutations could help identify patients at higher risk of recurrence, providing a basis for treatment decisions.

Drug sensitivity analysis evaluates the therapeutic effects of drugs on specific cells or tissues, guiding clinical treatment and precision medicine. In LUAD, CHRDL1 negatively correlates with the IC50 values of 40 drugs, with significant drugs including Damamerd and SB216763. Furthermore, CHRDL1 shows a positive correlation with the target gene PDGFRA of Avapritinib. These drugs play important roles in various signaling pathways. Damamerd, for instance, primarily acts by inhibiting AMPK and BMP signaling, regulating cell metabolism and development (Lin et al., 2023). PDGFRA, a tyrosine kinase receptor, activates PI3K/Akt, RAS/MAPK, and JAK/STAT pathways, promoting cell proliferation and survival (Maleddu et al., 2011). Avapritinib targets PDGFRA, inhibiting tumor cell growth (Dhillon, 2020). The potential effects of these drugs on CHRDL1 require further experimental validation.

Our study found that CHRDL1 is associated with immune cell infiltration, particularly showing strong positive correlations with endothelial cells and CD4^+^ T cells in LUAD. Moreover, CHRDL1 expression is significantly correlated with MHC-related molecules, suggesting that CHRDL1 may play a role in immune regulation, inflammatory responses, or antigen presentation. In LUAD, CHRDL1 positively correlates with several immune checkpoint molecules, including CD44, CD48, and CD86, and negatively correlates with TNFRSF25 and TNFRSF18. CHRDL1 is positively correlated with the tumor immune cycle, while negatively correlated with base excision repair, cell cycle, and DNA replication. These findings suggest that CHRDL1 may participate in multiple immune cycle steps, and targeting CHRDL1 may inhibit tumor growth by affecting the cell cycle and DNA replication. The role of CHRDL1 in the immune environment of LUAD needs further investigation.

We observed that CHRDL1 functions as a tumor suppressor in LUAD by inhibiting cell growth and proliferation. Studies on the role of CHRDL1 in regulating cell growth and proliferation are rare. One study showed that CHRDL1 inhibits the proliferation and migration of mesenchymal stem cells derived from amniotic fluid (Huang et al., 2020). Pei et al. found through *in vitro* experiments in gastric cancer cells that CHRDL1 knockdown promotes tumor cell proliferation and migration (Pei et al., 2017). The mechanism by which CHRDL1 inhibits LUAD cell proliferation remains to be explored.

Notably, we found that CHRDL1 significantly inhibits LUAD invasion and metastasis by downregulating the TGF-β pathway. Transcriptome sequencing results showed that high expression of CHRDL1 significantly downregulates the transcription levels of TGF-β-related downstream target genes (e.g., SNAIL1, TGM2, JUNB), while the transcription levels of Smad2/3 and TGFBR1/2 remain unchanged (Supplementary Figure S6A). Immunofluorescence results showed that knockdown of CHRDL1 promotes the nuclear translocation of pSmad2, suggesting that CHRDL1 may antagonize TGF-β, reducing the nuclear translocation of pSmad2/3, thereby inhibiting EMT mediated by the TGF-β pathway (Supplementary Figure S6B). Previous research has shown that CHRDL1 can bind to TGF-β in cardiac tissue to inhibit fibrosis and maladaptive cardiac remodeling (Ruozi et al., 2022). In ovarian tissue, CHRDL1 works synergistically with TWSG1 to antagonize members of the TGF-β superfamily (Wang et al., 2021). Li et al. confirmed that CHRDL1 suppresses colorectal cancer metastasis by regulating the EMT pathway. In breast cancer, CHRDL1 inhibits BMP4-induced cell migration (Cyr-Depauw et al., 2016). These findings suggest that CHRDL1 exerts its function by antagonizing BMP and TGF-β signaling. Our study further explores the potential of CHRDL1 as a therapeutic target in LUAD and the underlying mechanisms.

In conclusion, this study, through comprehensive bioinformatics analysis and experimental validation, depicts the expression patterns of CHRDL1 in various cancers, particularly emphasizing its role in LUAD. Our findings reveal significant correlations between CHRDL1 expression, patient survival outcomes, immune cell infiltration, and drug responses. Specifically, in LUAD, high CHRDL1 expression is associated with better prognosis and exerts its inhibitory effect through the TGF-β pathway by suppressing EMT. These insights not only highlight the multifaceted impact of CHRDL1 in tumorigenesis but also underscore its potential as a therapeutic target in LUAD, paving the way for advancements in personalized medicine and immunotherapy. A limitation of this study is the lack of in-depth exploration of the regulatory role of CHRDL1, including its relationship with immune regulation and its therapeutic effects. Additionally, the use of a single shRNA for gene knockdown in our experiments may carry a risk of “off-target effects”, which should be minimized.

## Data Availability

Publicly available datasets were analyzed in this study. This data can be found here: https://portal.gdc.cancer.gov; http://commonfund.nih.gov/GTEx/; https://discover.nci.nih.gov/cellminer/home.do.

## Glossary

LUAD: Lung adenocarcinoma
TCGA: The Cancer Genome Atlas
GTEx: Genotype-Tissue Expression Project
CHRDL1: Chordin-Like 1
TME: Tumor microenvironment
NSCLC: Non-small cell lung cancer
BLCA: Bladder urothelial carcinoma
GEO: Gene Expression Omnibus
ICGC: International Cancer Genome Consortium
OS: Overall survival
RFS: Recurrence free survival
GEPIA: Gene expression profiling interactive analysis
HR: Hazard ratio
DSS: Disease specific survival
CI: Confidence interval
OV: Ovarian cancer
STAD: Stomach adenocarcinoma
LIHC: Liver hepatocellular carcinoma
K-M: Kaplan-Meier
PFS: Progression free survival
FP: First progression
PPS: Post progression survival
FDA: Food and drug administration
**IC**_**50**_: Half-maximal inhibitory concentrations
TTD: Therapeutic Target Database
BRCA: Breast invasive carcinoma
CESC: Cervical squamous cell carcinoma
COAD: Colon adenocarcinoma
SKCM: Skin cutaneous melanoma
