# Supplementary material for: Pan-cancer analysis of CHRDL1 expression and its mechanistic role in inhibiting EMT via the TGF-β pathway in lung adenocarcinoma
Source: Front Cell Dev Biol. 2025 Mar 31;13:1557761. doi: 10.3389/fcell.2025.1557761 (PMC11994622; doi:10.3389/fcell.2025.1557761)
Supplement: Supplementary file 2 [file DataSheet2.zip › Supplementary figures and tables/Supplementary Figures Final.docx]

**Supplementary Fig 1.**
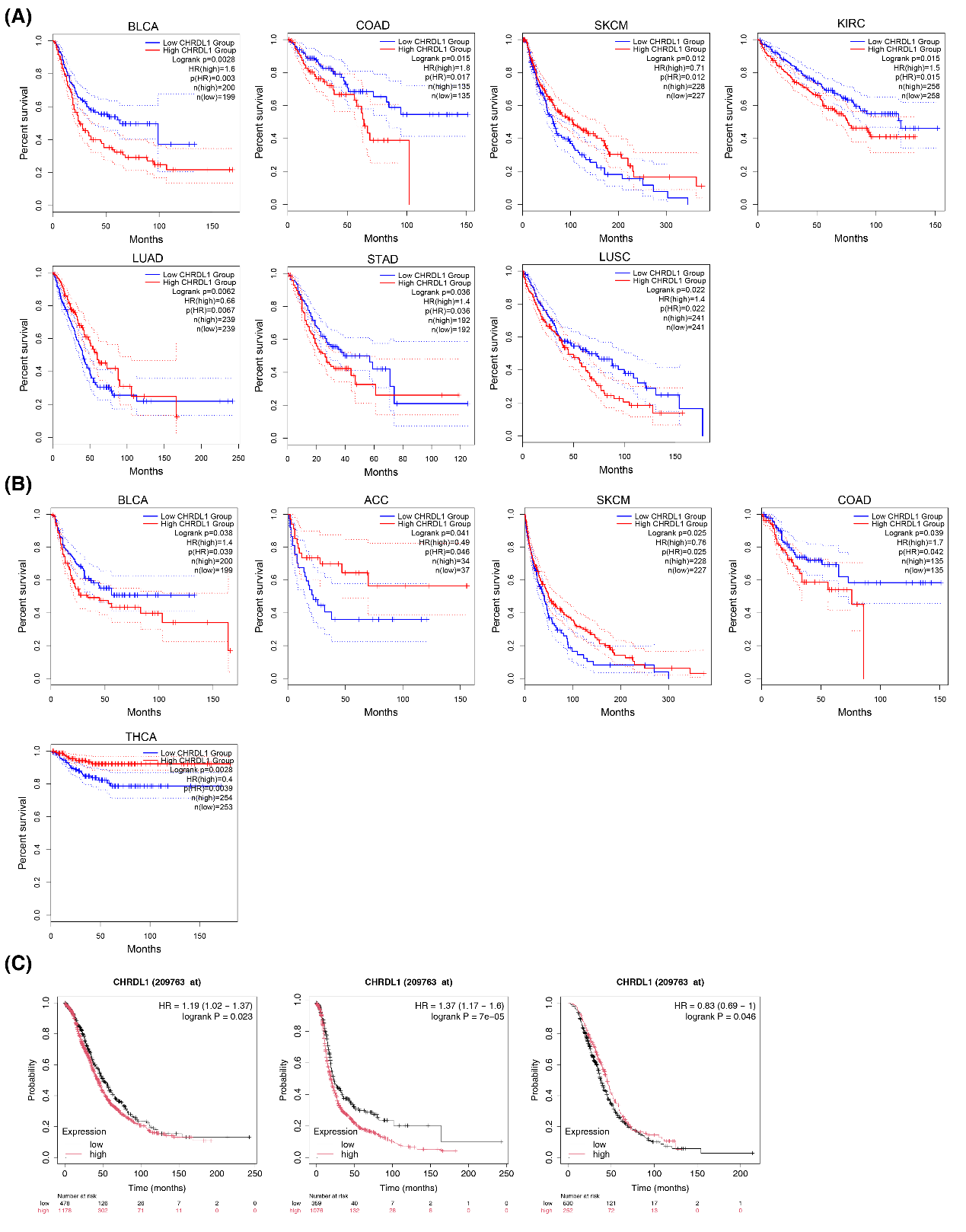
 OS **(A)** and RFS **(B)** with high and low expression of CHRDL1 in various cancers. (**C)** The OS, PFS and PPS of OV with different expression of CHRDL1.

**Supplementary Fig**
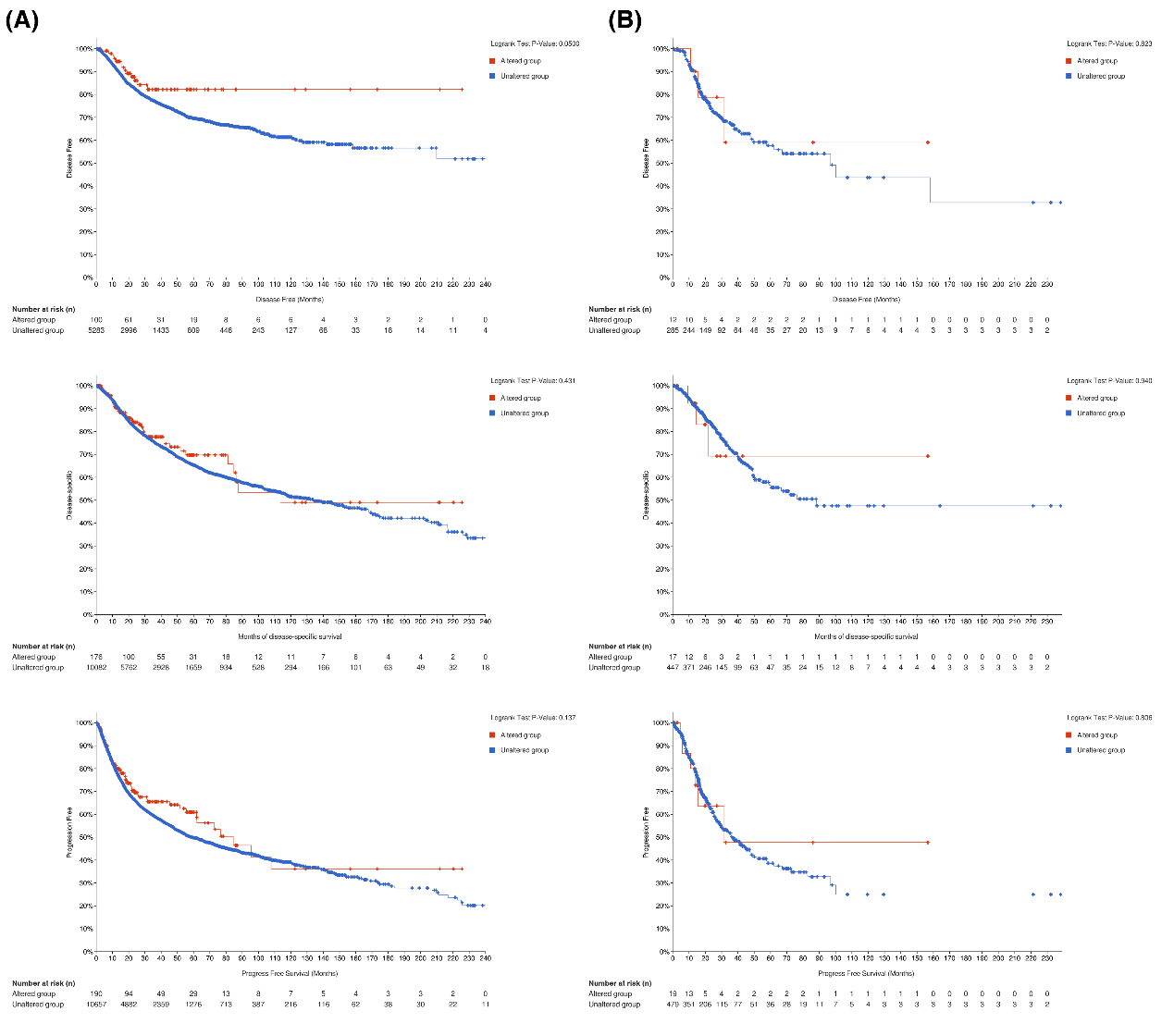
**2.** (**A**) DFS, DSS, and PFS between patients with and without CHRDL1 mutation in pan-cancer (p > 0.05). **(B)** DFS, DSS, and PFS between patients with and without CHRDL1 mutation in LUAD (p > 0.05).


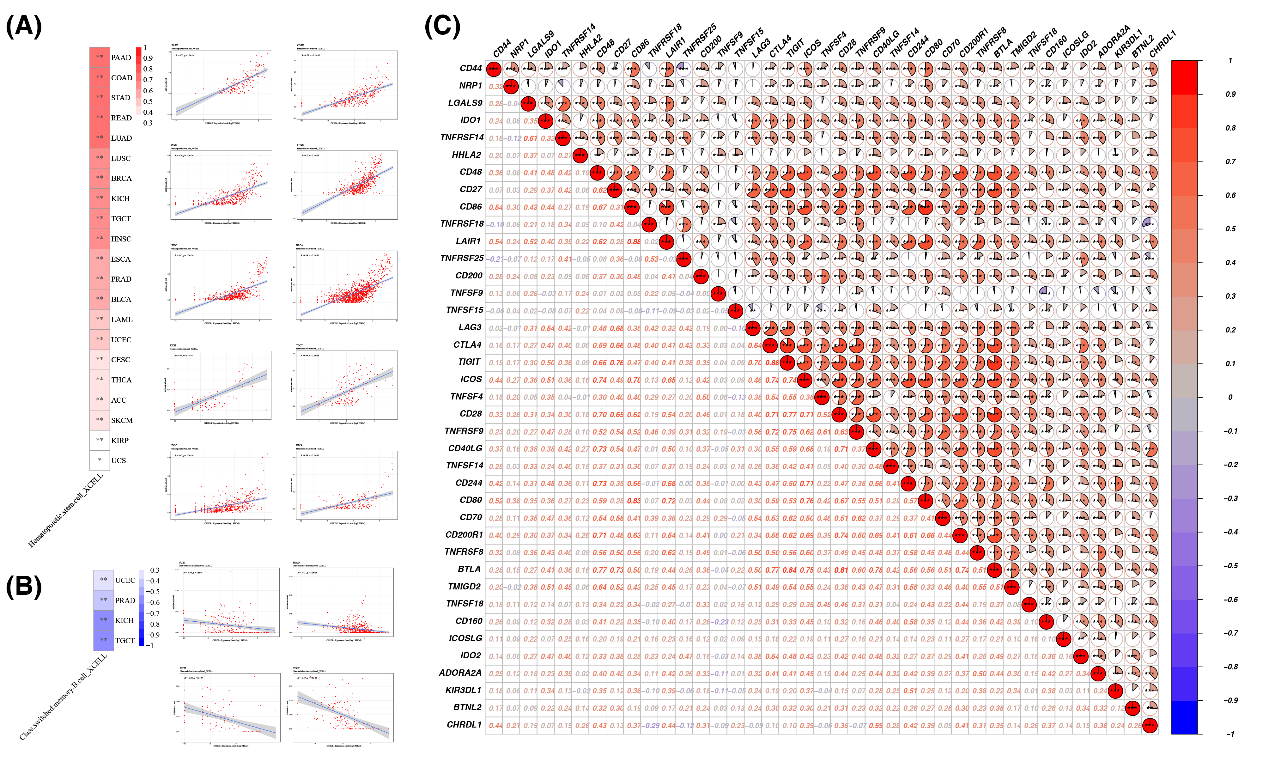
**Supplementary Fig 3.** Cancers with positive correlation(**A**) and negative correlation (**B**) between immune cell infiltration and CHRDL1 expression in pan-cancer. (**C**) Correlation of CHRDL1 expression with and immune checkpoint genes in Pan-Cancer.

**Supplementary Fig**
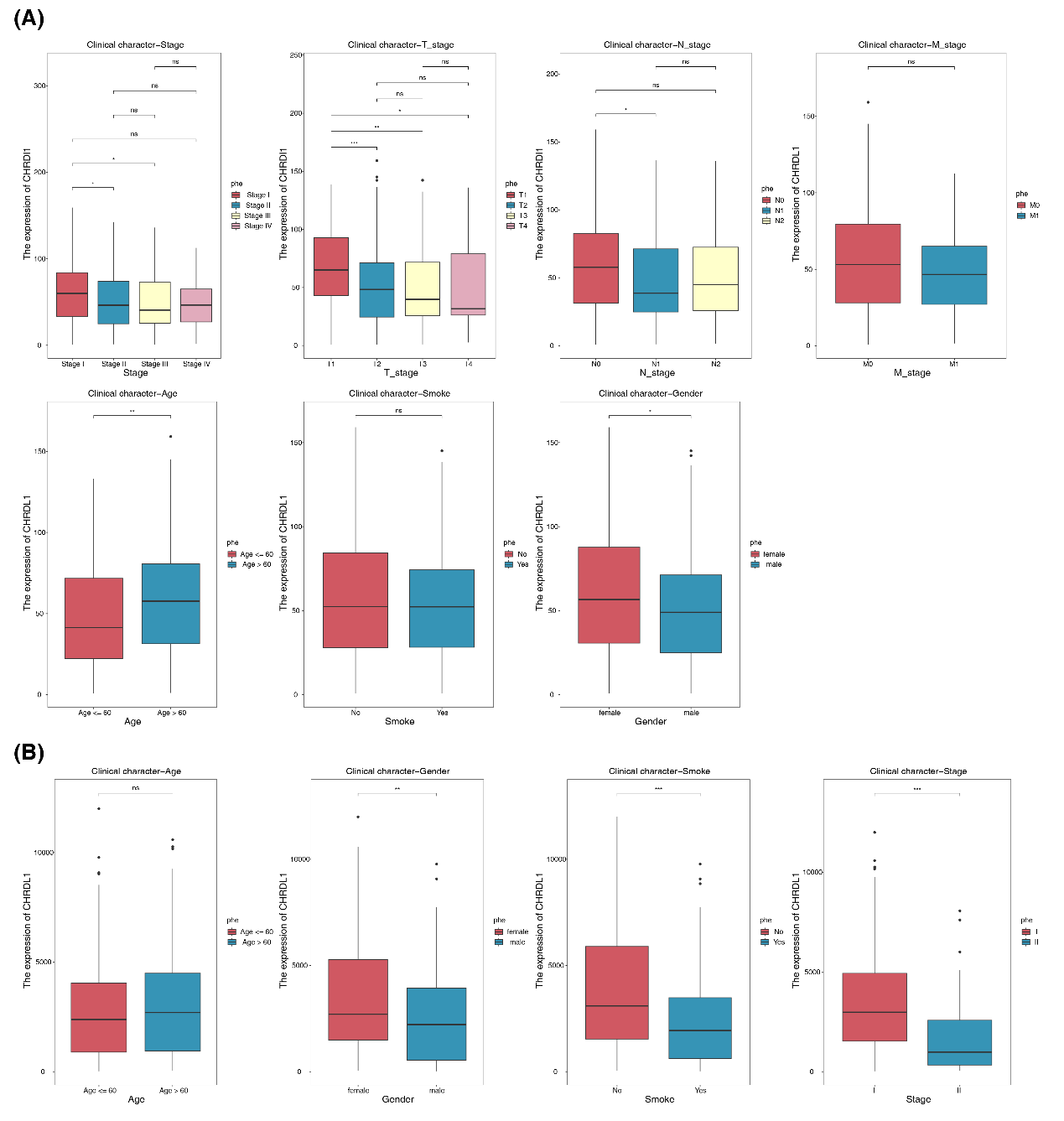
**4.** Expression of CHRDL1 across various clinical features in TCGA (**A**) and GSE31210 (**B**) cohorts.


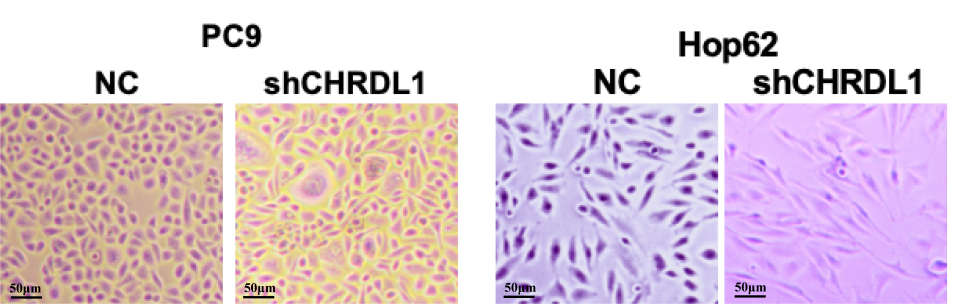
**Supplementary Fig 5.** The picture of shCHRDL1 cells vs NC.


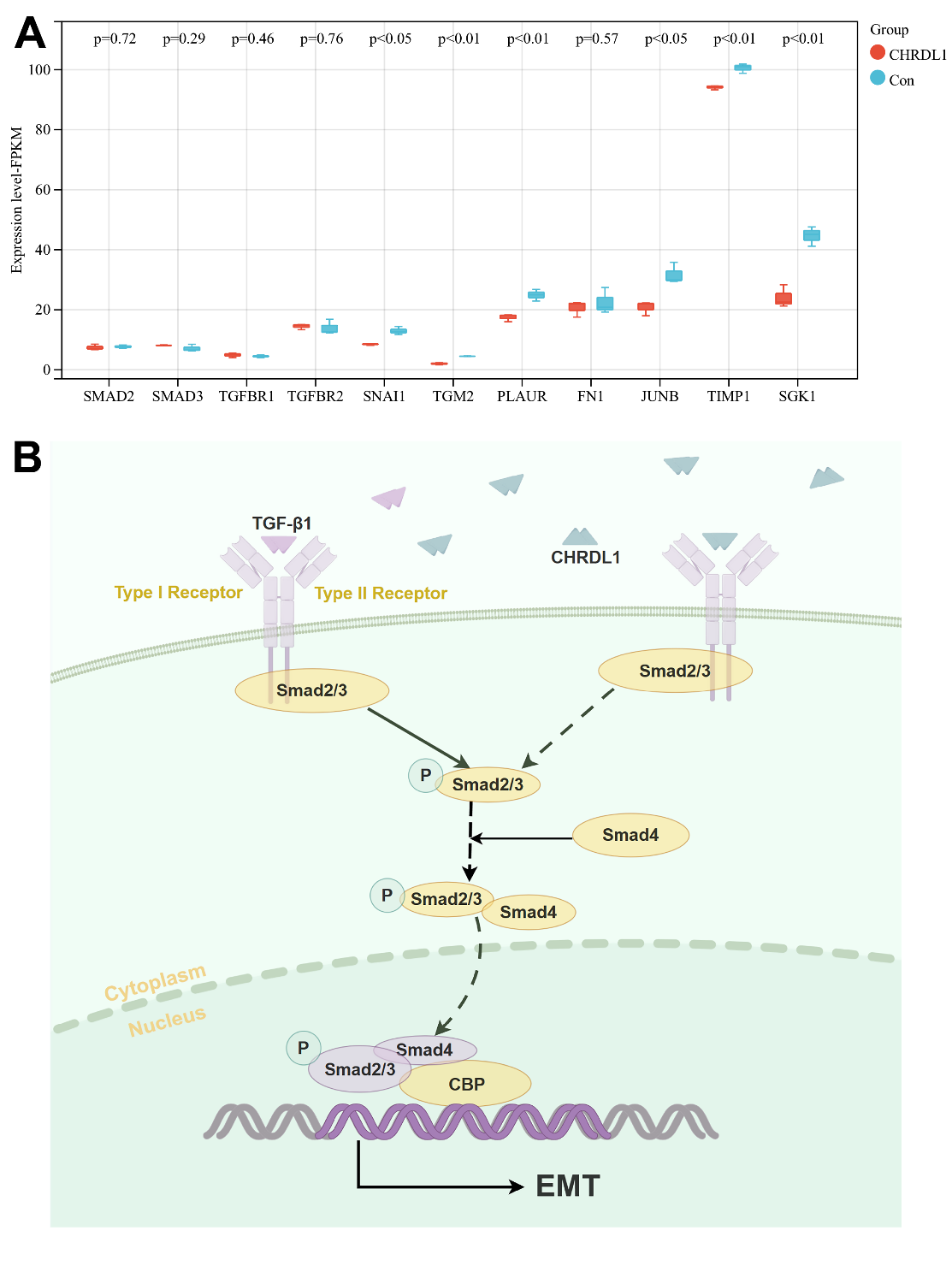
**Supplementary Fig6.** **(A)** Comparison of the expression levels of TGF-β pathway-related genes between cells with high CHRDL1 expression and normal cells. **(B)** The mechanism by which CHRDL1 regulates the TGF-β pathway in LUAD. This image was created online by Figdraw.
