# Supplementary material for: Pan-cancer analysis of CHRDL1 expression and its mechanistic role in inhibiting EMT via the TGF-β pathway in lung adenocarcinoma
Source: Front Cell Dev Biol. 2025 Mar 31;13:1557761. doi: 10.3389/fcell.2025.1557761 (PMC11994622; doi:10.3389/fcell.2025.1557761)

Figure 8A

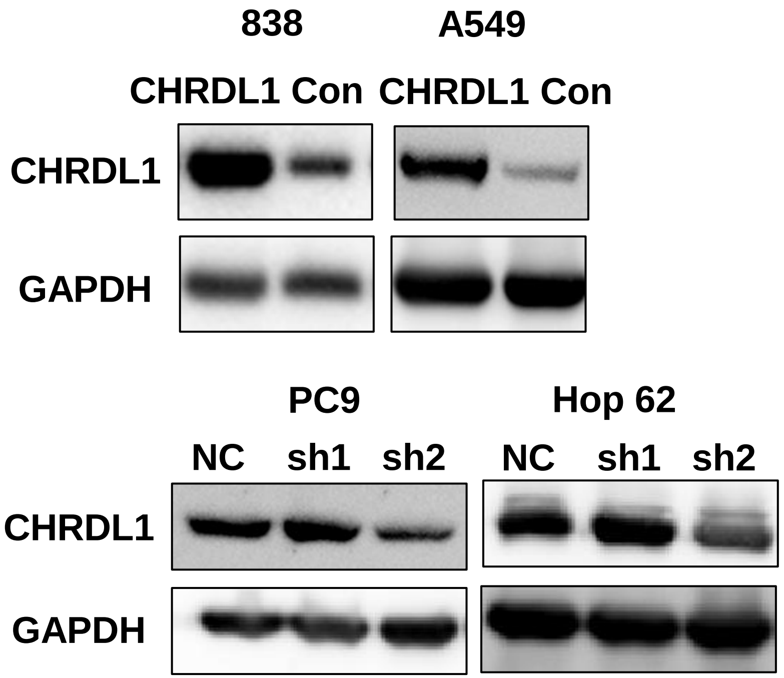

838
A549
CHRDL1 Con
CHRDL1 Con
CHRDL1
GAPDH
PC9
Hop 62
NC
sh1
sh2
NC
sh1
sh2
CHRDL1
GAPDH

838-CHRDL1

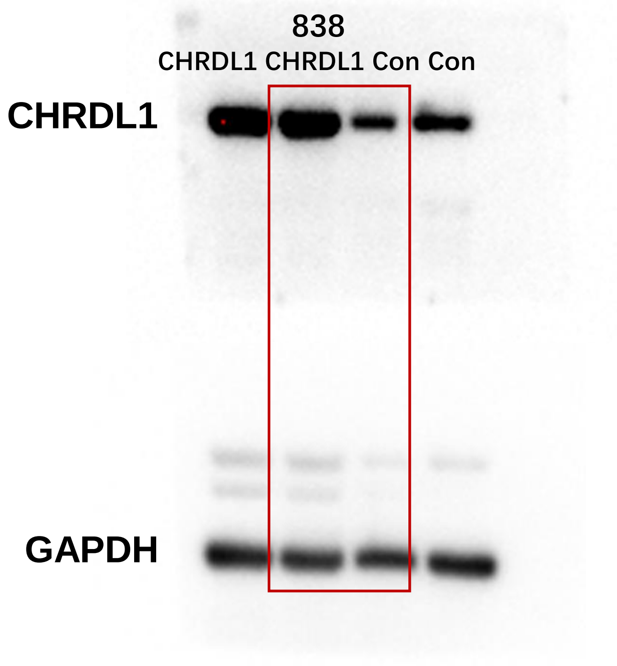
838
CHRDL1 CHRDL1 Con Con
CHRDL1
GAPDH

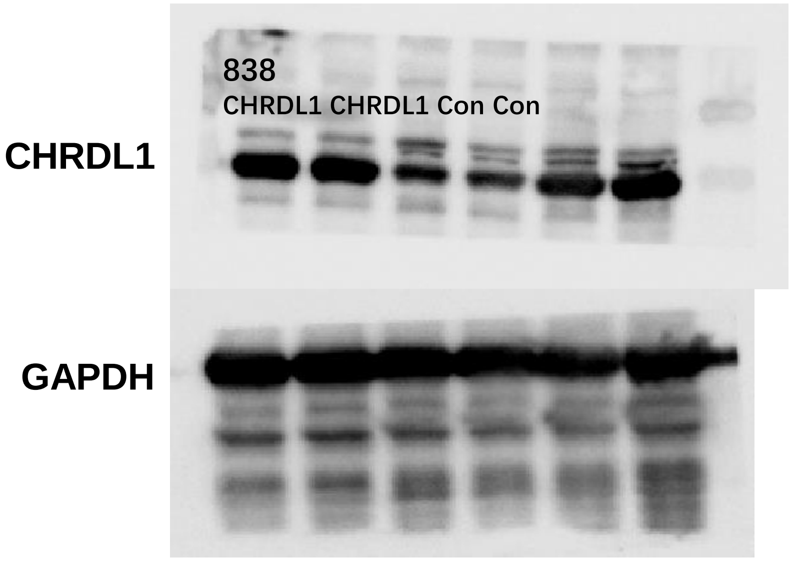
838
CHRDL1 CHRDL1 Con Con
CHRDL1
GAPDH

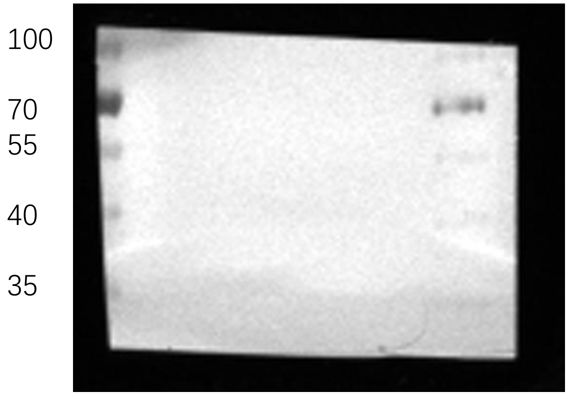
100
70
55
40
35

A549-CHRDL1

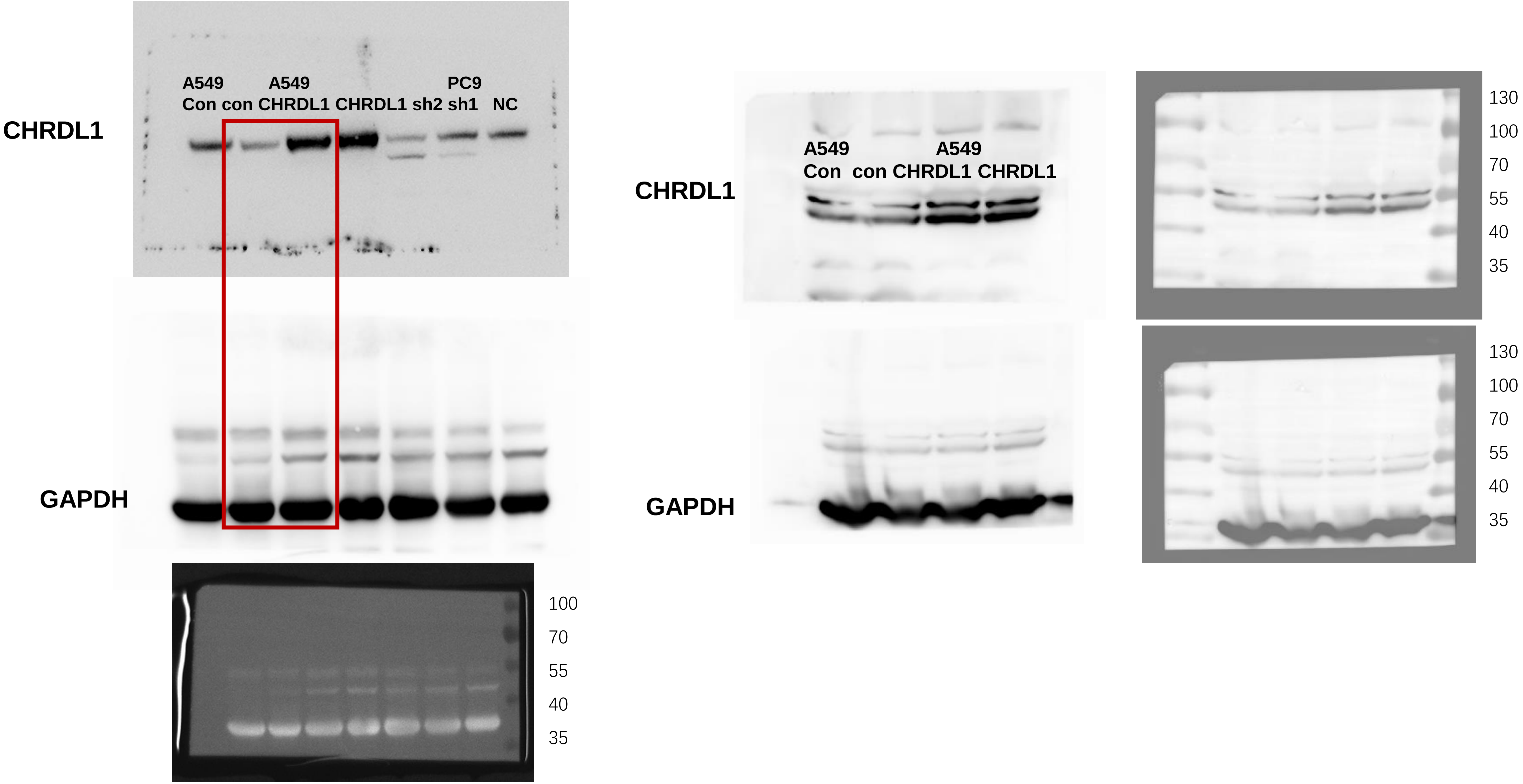

CHRDL1
A549
A549
PC9
Con con CHRDL1 CHRDL1 sh2 sh1 NC
GAPDH
100
70
55
40
35
CHRDL1
A549
A549
Con con CHRDL1 CHRDL1
GAPDH
130
100
70
55
40
35
130
100
70
55
40
35

PC9-CHRDL1

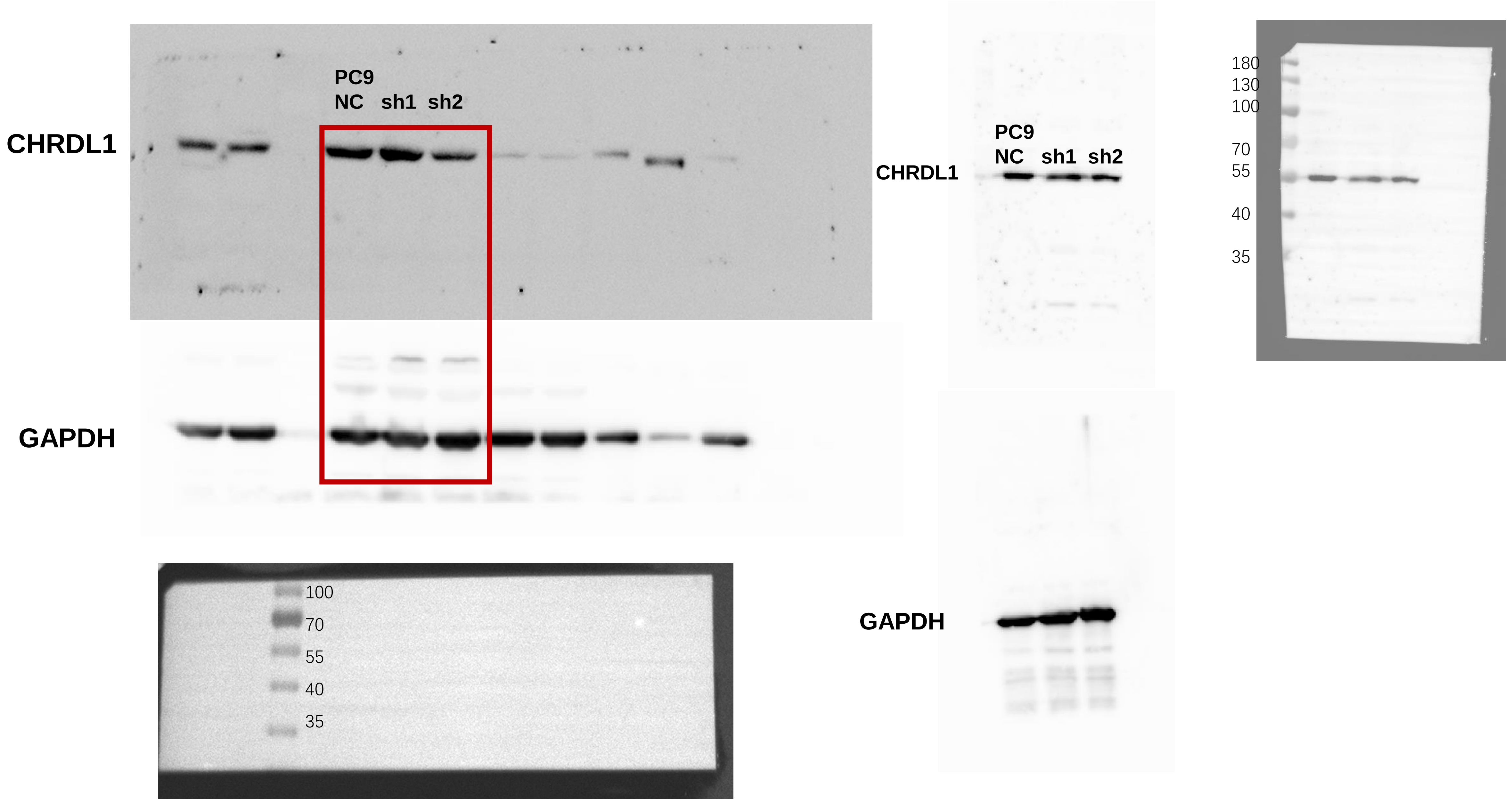
CHRDL1
PC9
NC sh1 sh2
GAPDH
100
70
55
40
35
CHRDL1
PC9
NC sh1 sh2
GAPDH
180
130
100
70
55
40
35

Hop 62-CHRDL1

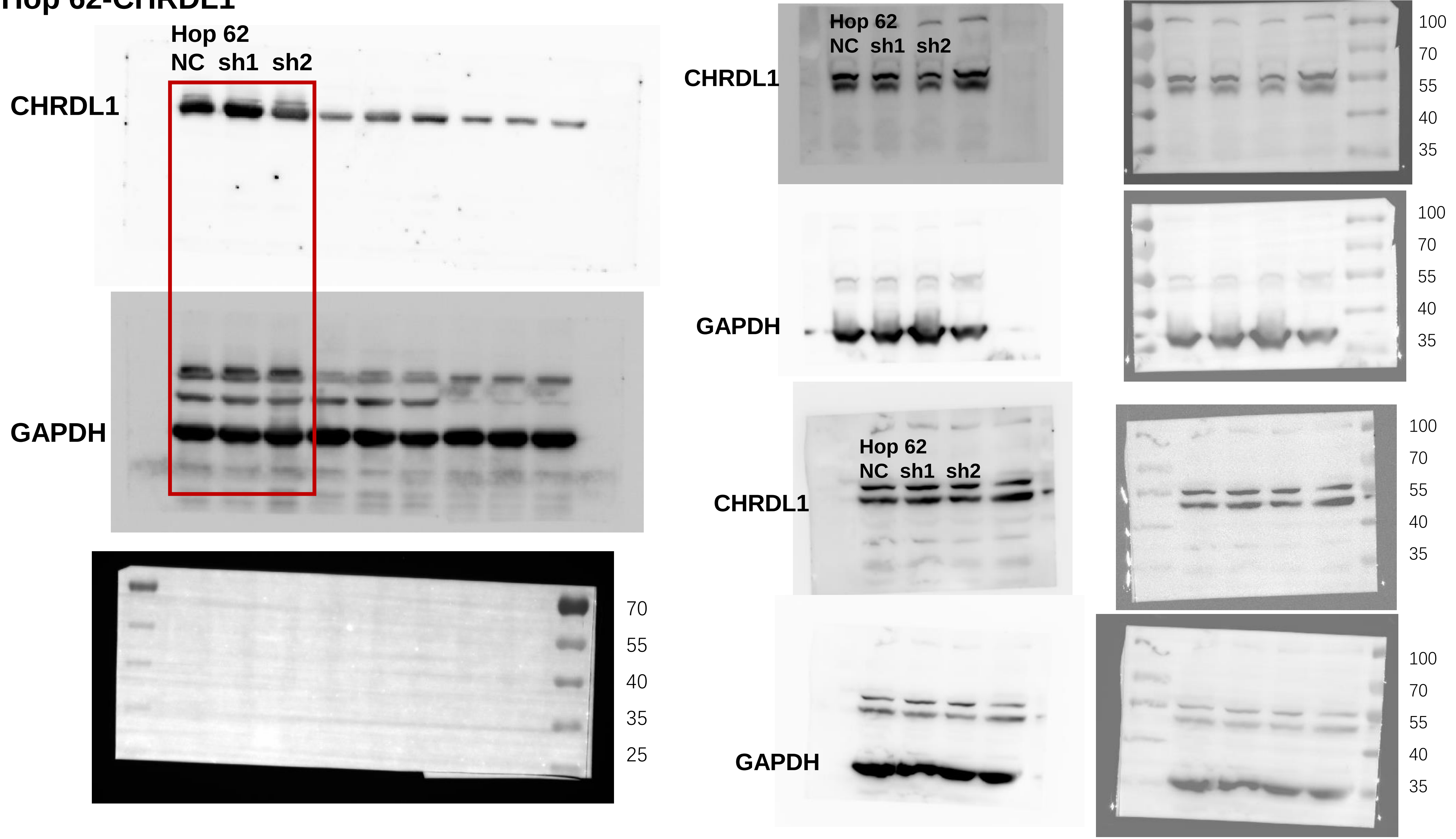

Figure 9C

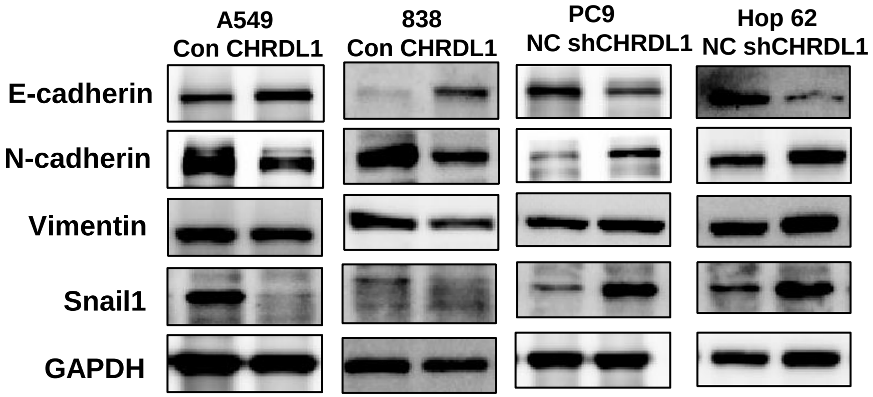
A549
Con CHRDL1
838
Con CHRDL1
PC9
NC shCHRDL1
Hop 62
NC shCHRDL1
E-cadherin
N-cadherin
Vimentin
Snail1
GAPDH

A549

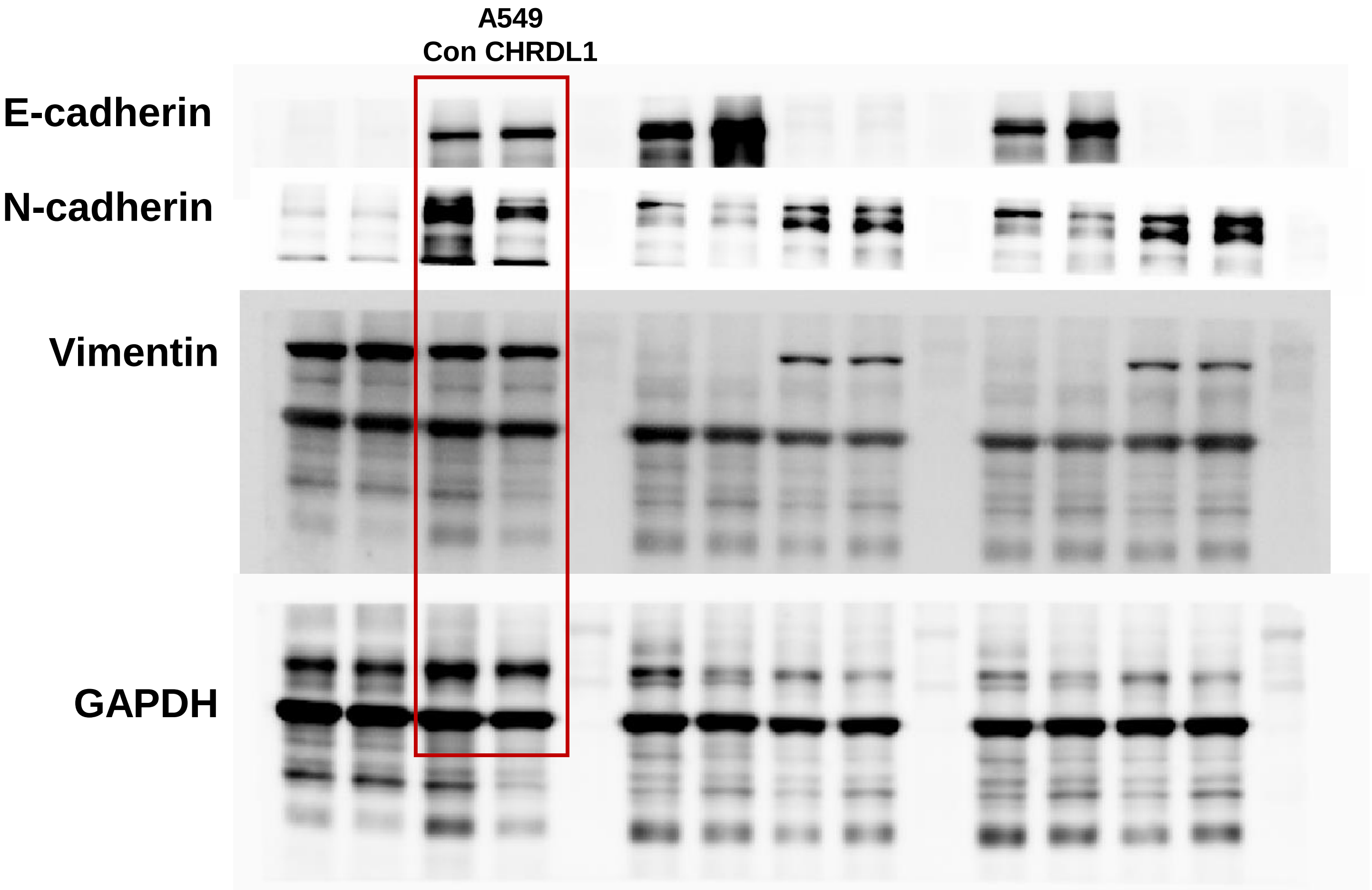
A549
Con CHRDL1
E-cadherin
N-cadherin
Vimentin
GAPDH

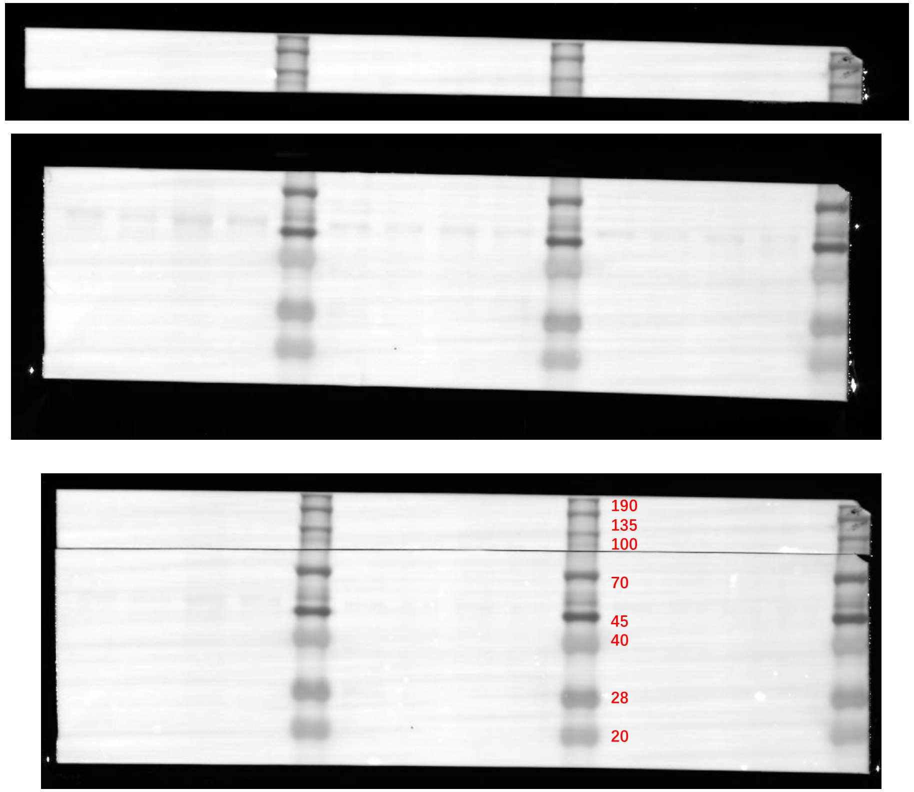
190
135
100
70
45
40
28
20

A549

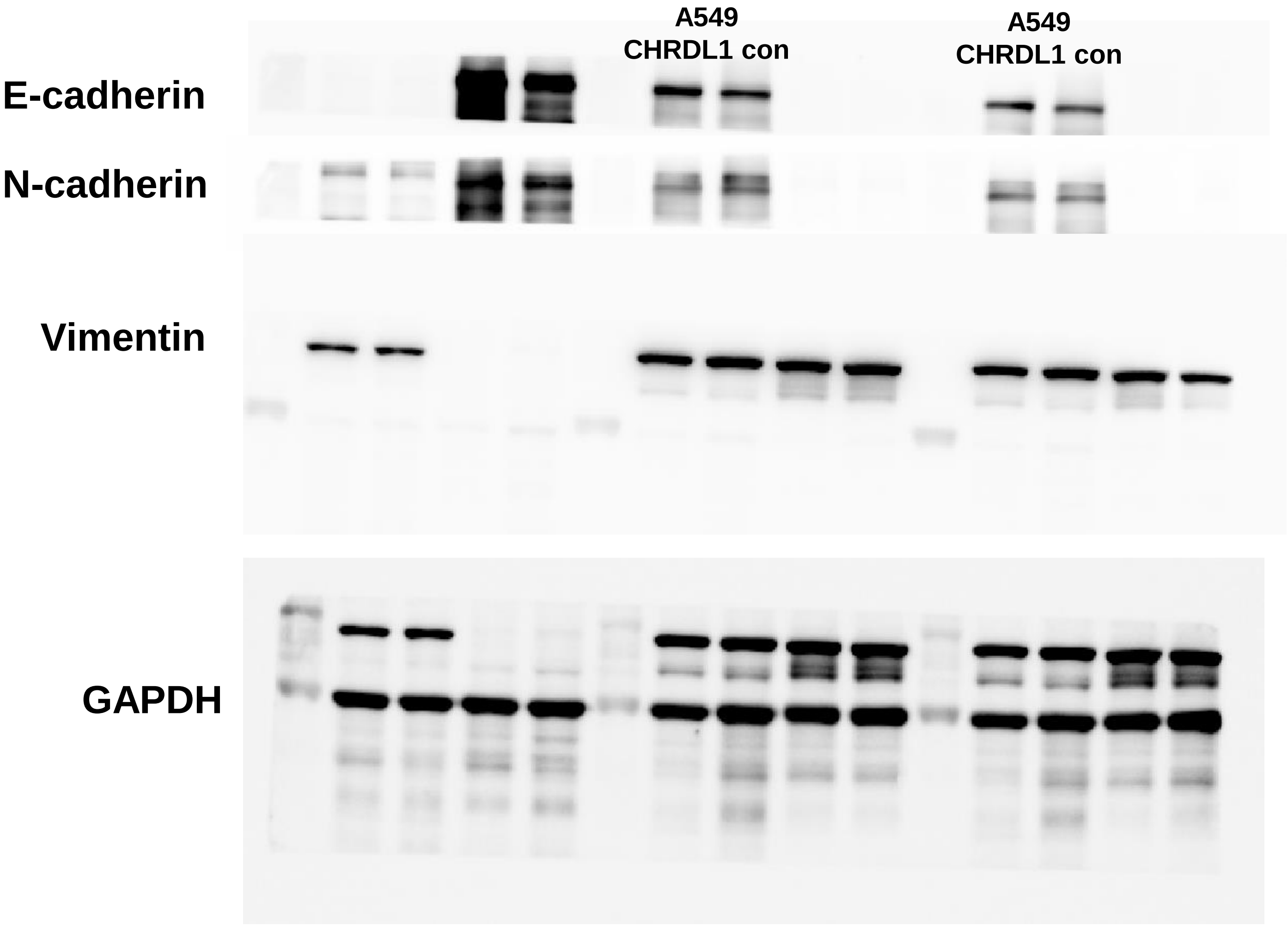
A549
CHRDL1 con
A549
CHRDL1 con
E-cadherin
N-cadherin
Vimentin
GAPDH

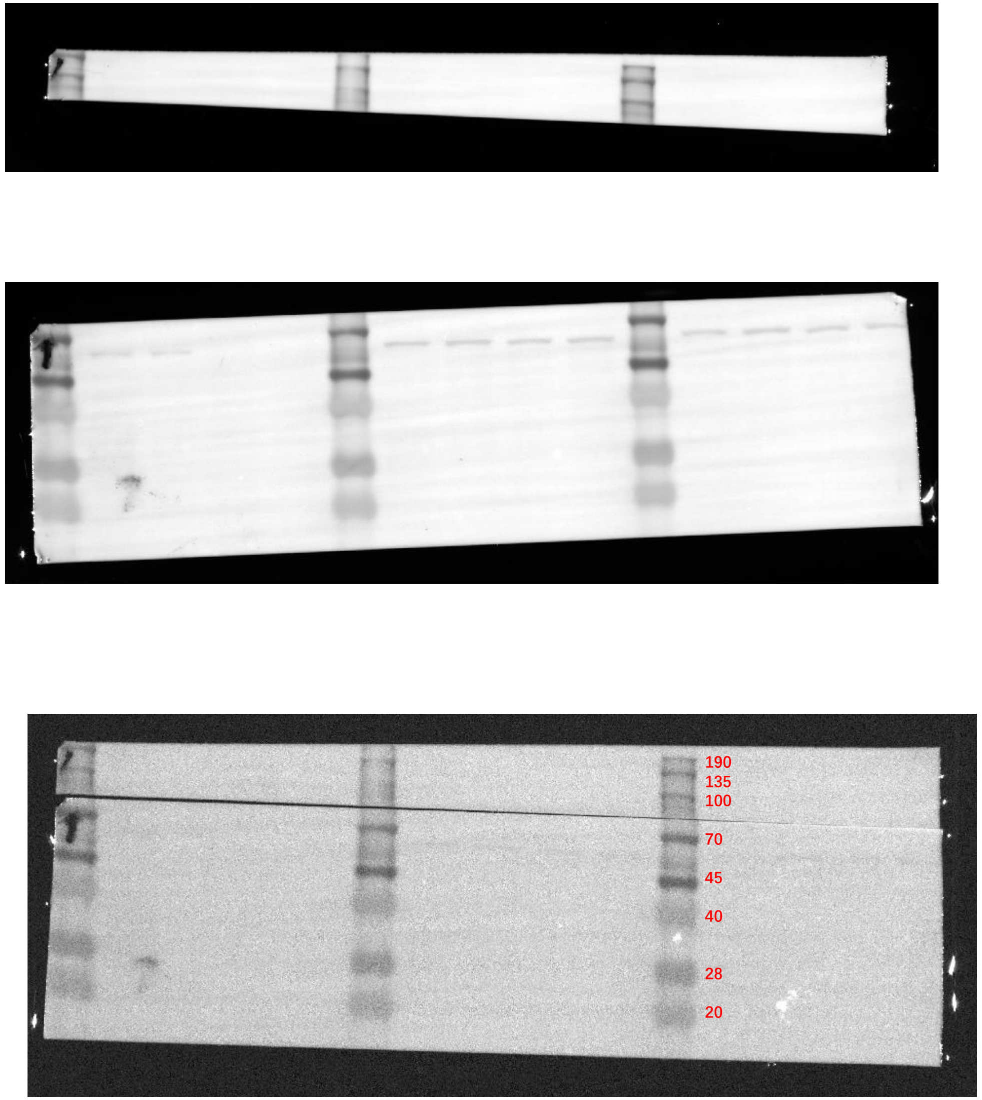
190
135
100
70
45
40
28
20

PC9

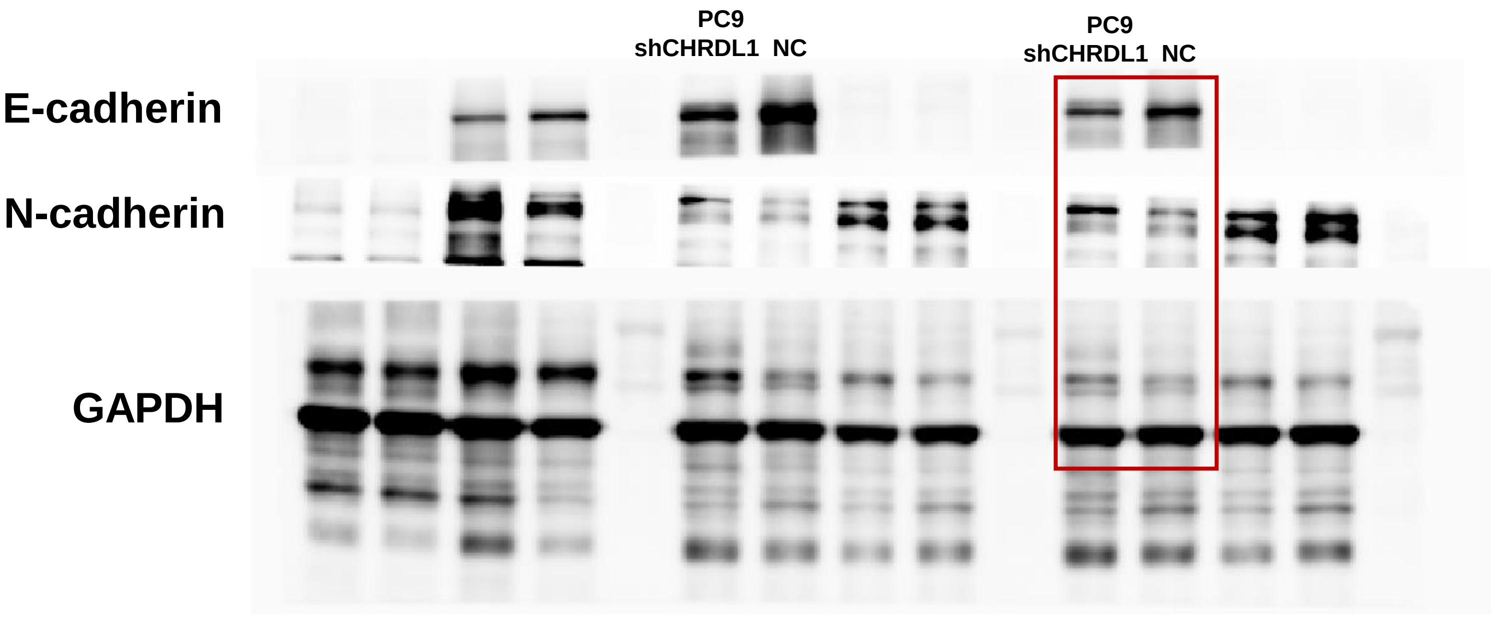
PC9
shCHRDL1 NC
PC9
shCHRDL1 NC
E-cadherin
N-cadherin
GAPDH

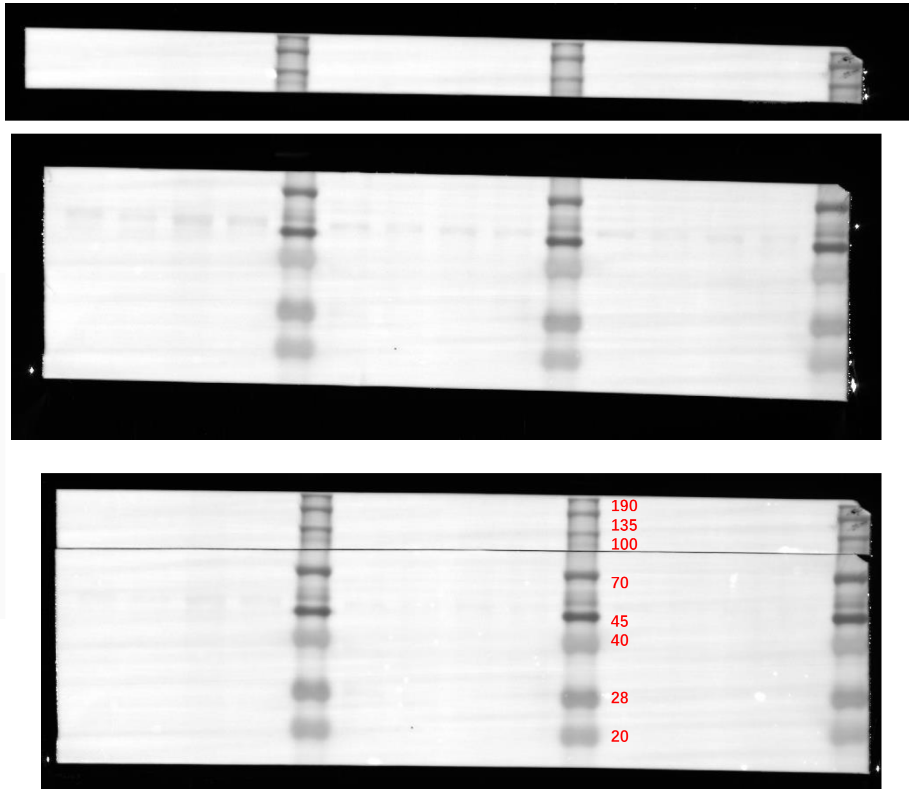
190
135
100
70
45
40
28
20

PC9

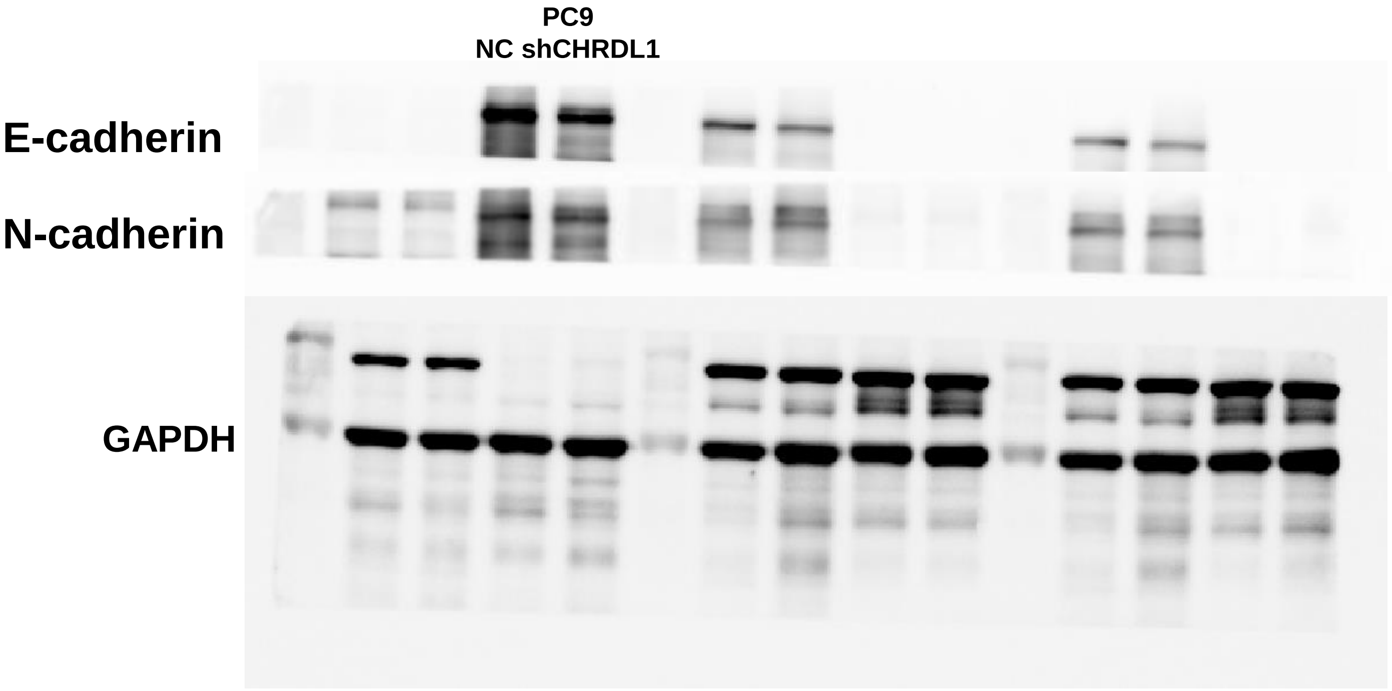
PC9
NC shCHRDL1
E-cadherin
N-cadherin
GAPDH

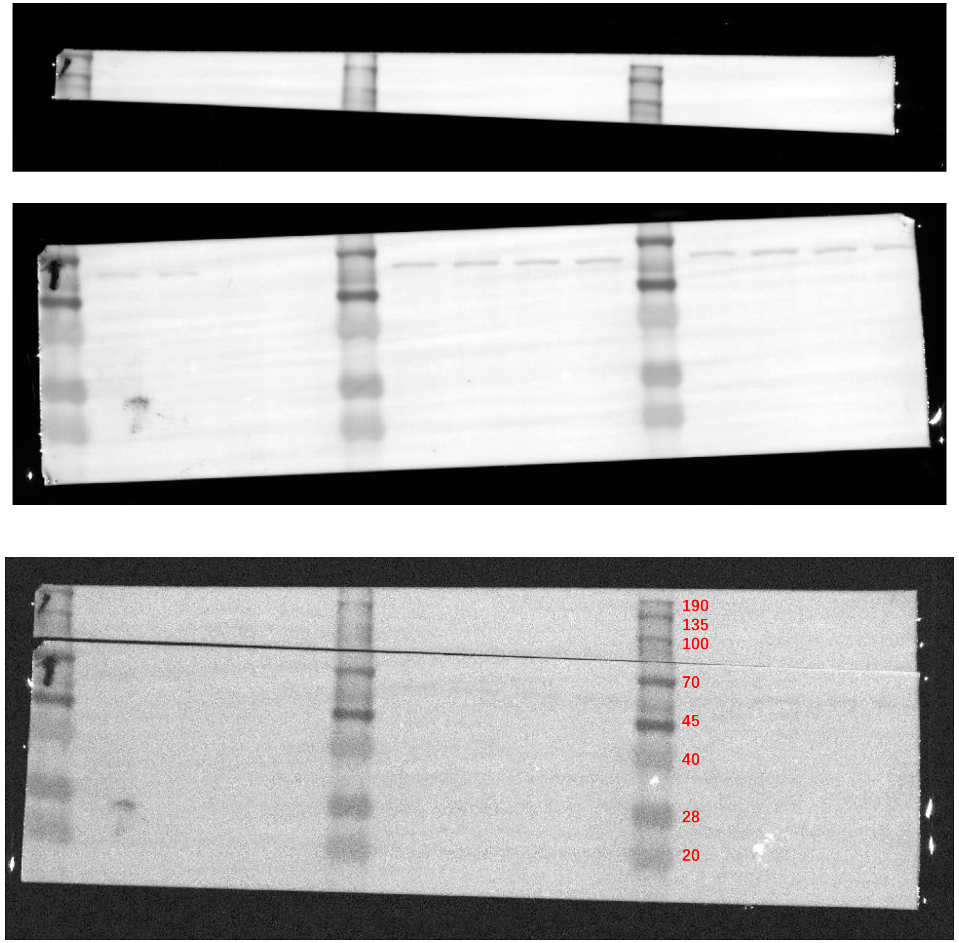
190
135
100
70
45
40
28
20

PC9-Vimentin

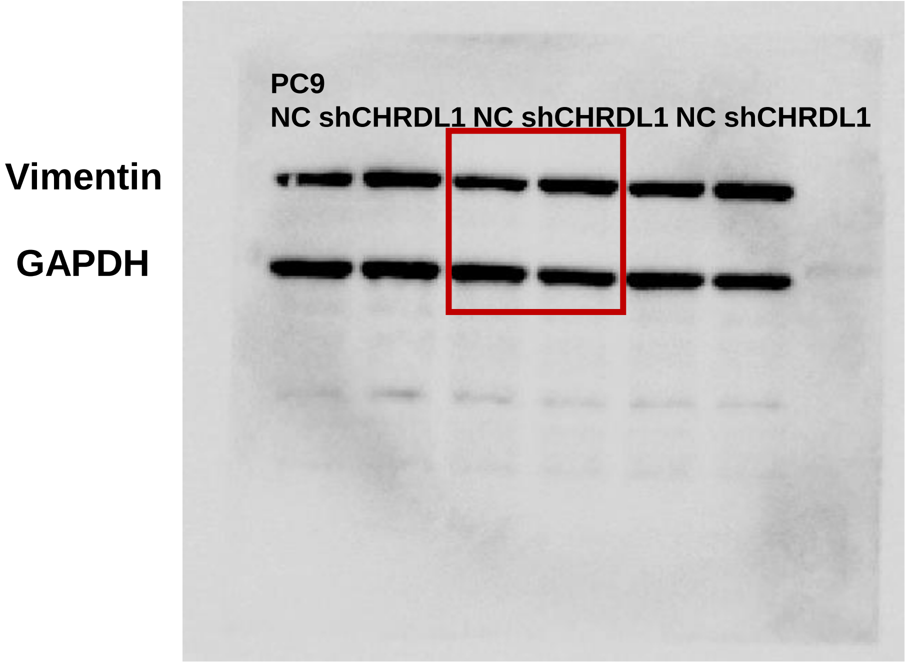

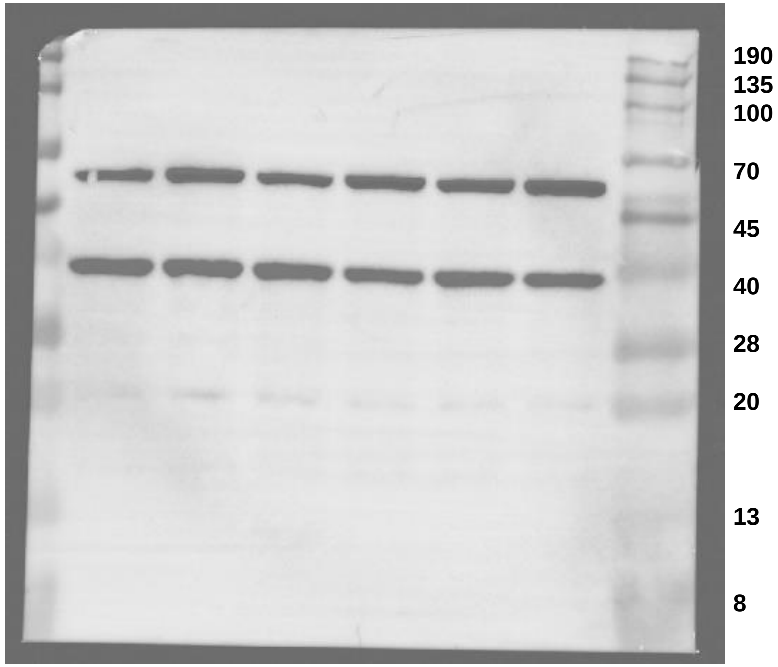

838

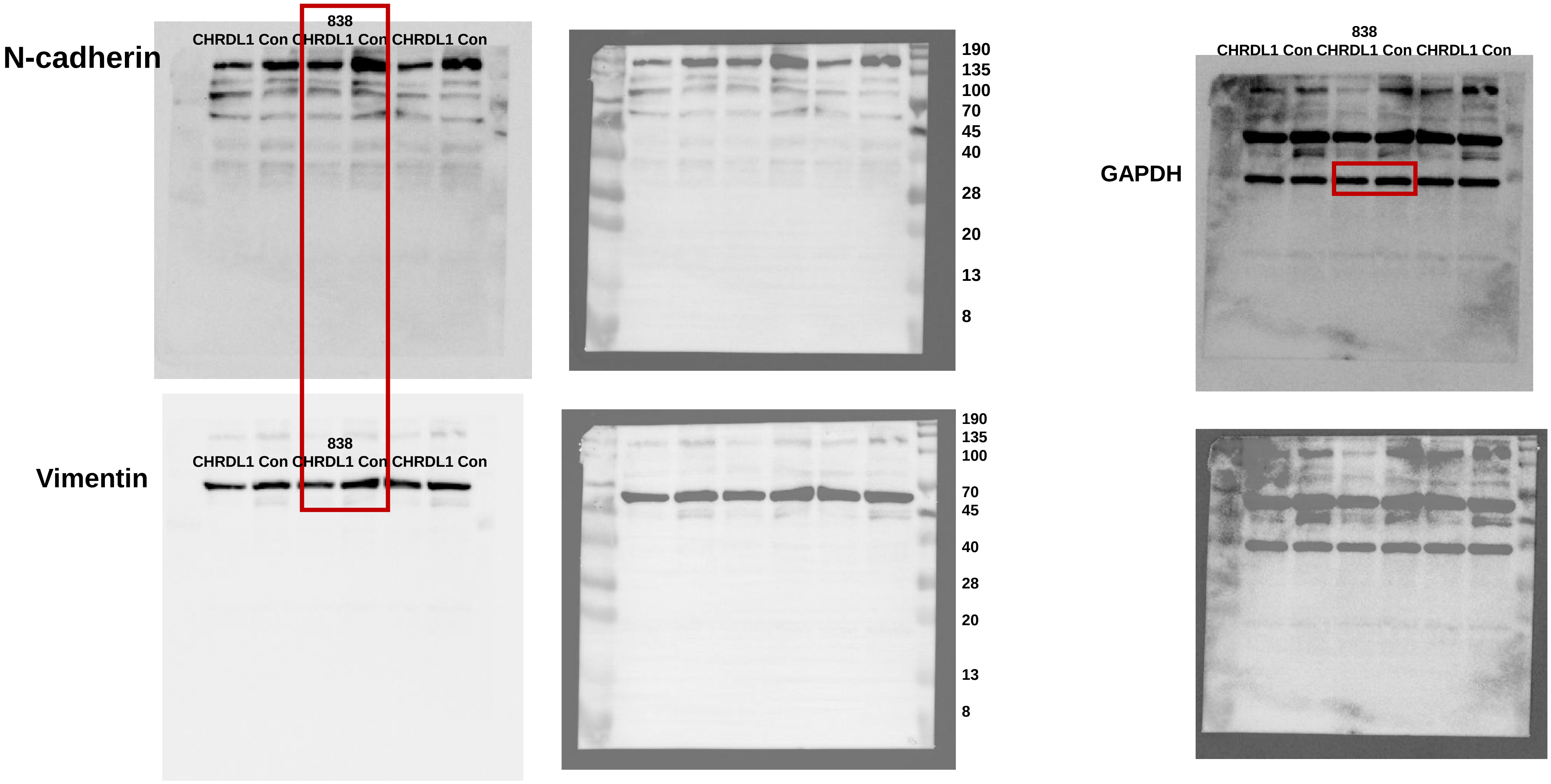
N-cadherin
838
CHRDL1 Con CHRDL1 Con CHRDL1 Con
190
135
100
70
45
40
28
20
13
8
Vimentin
838
CHRDL1 Con CHRDL1 Con CHRDL1 Con
190
135
100
70
45
40
28
20
13
8
GAPDH
838
CHRDL1 Con CHRDL1 Con CHRDL1 Con

838

E-cadherin

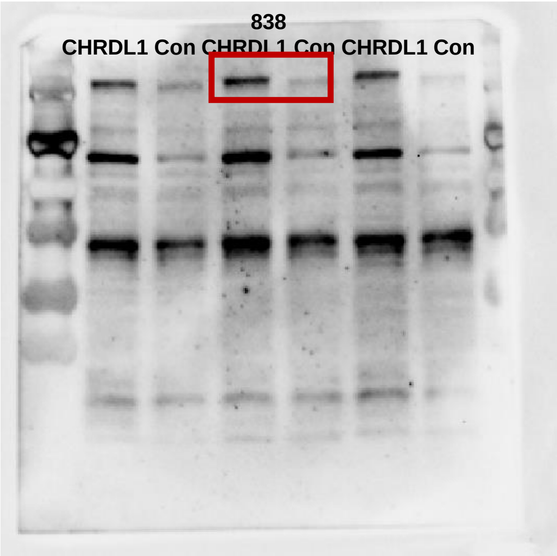

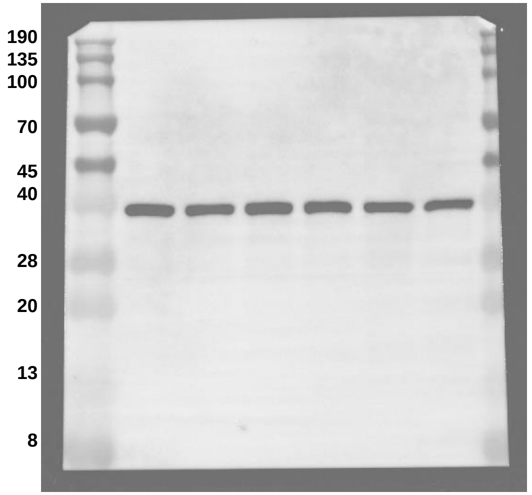

GAPDH

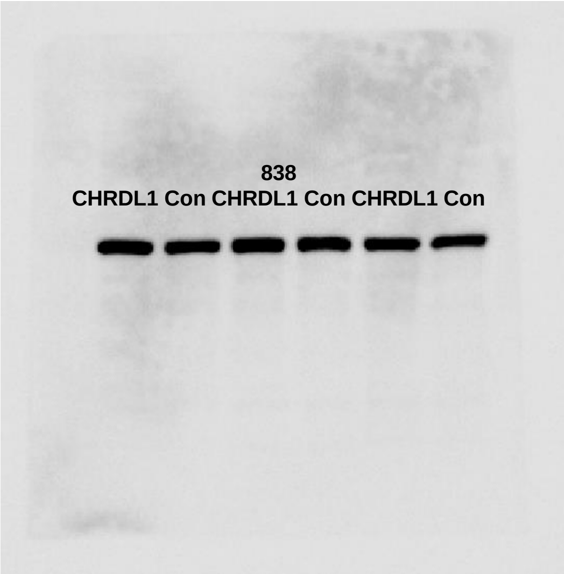

Hop62

N-cadherin

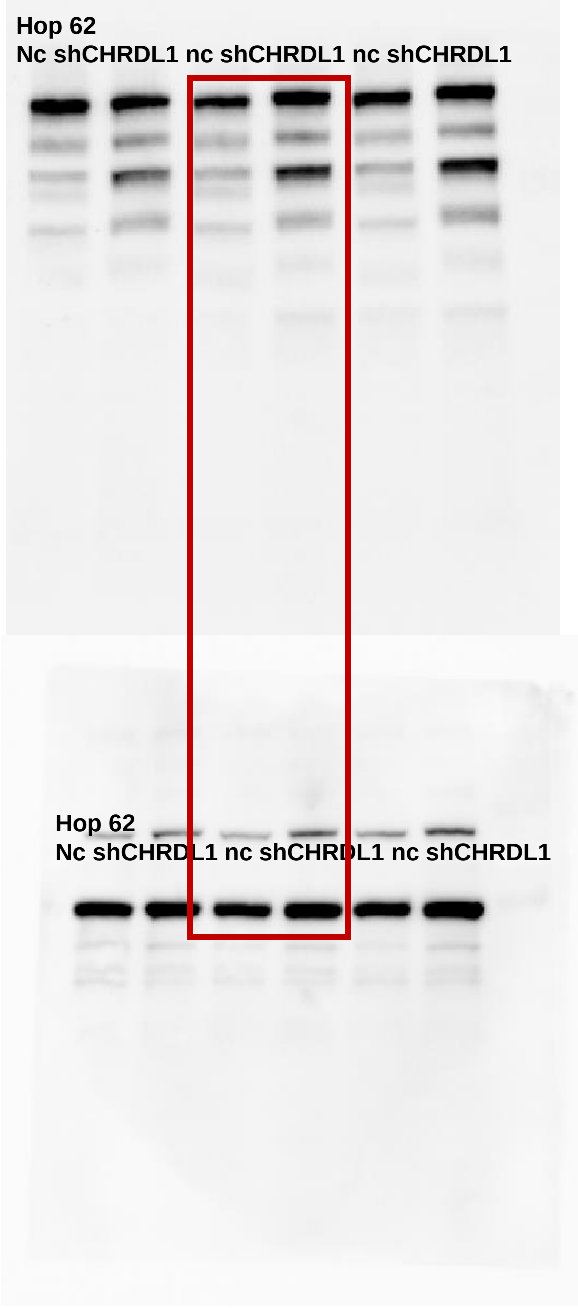

GAPDH

Vimentin

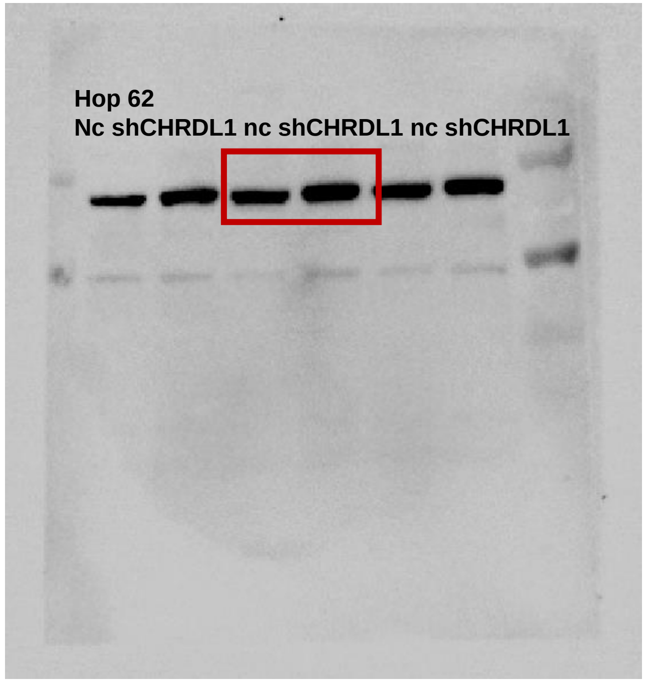

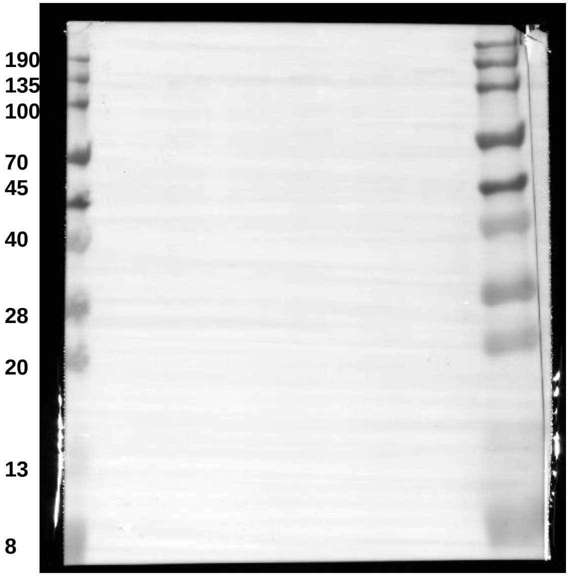

Hop62

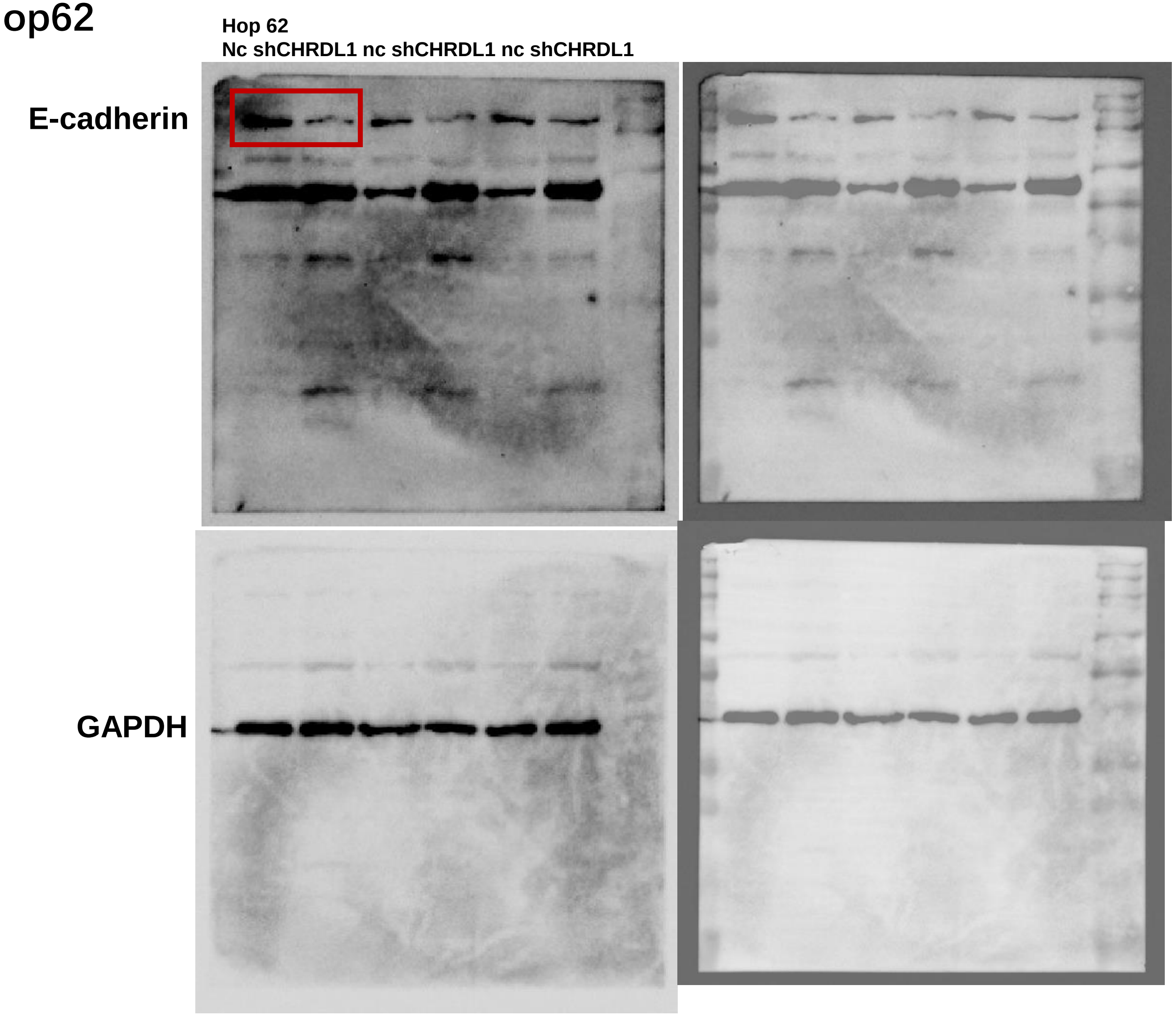

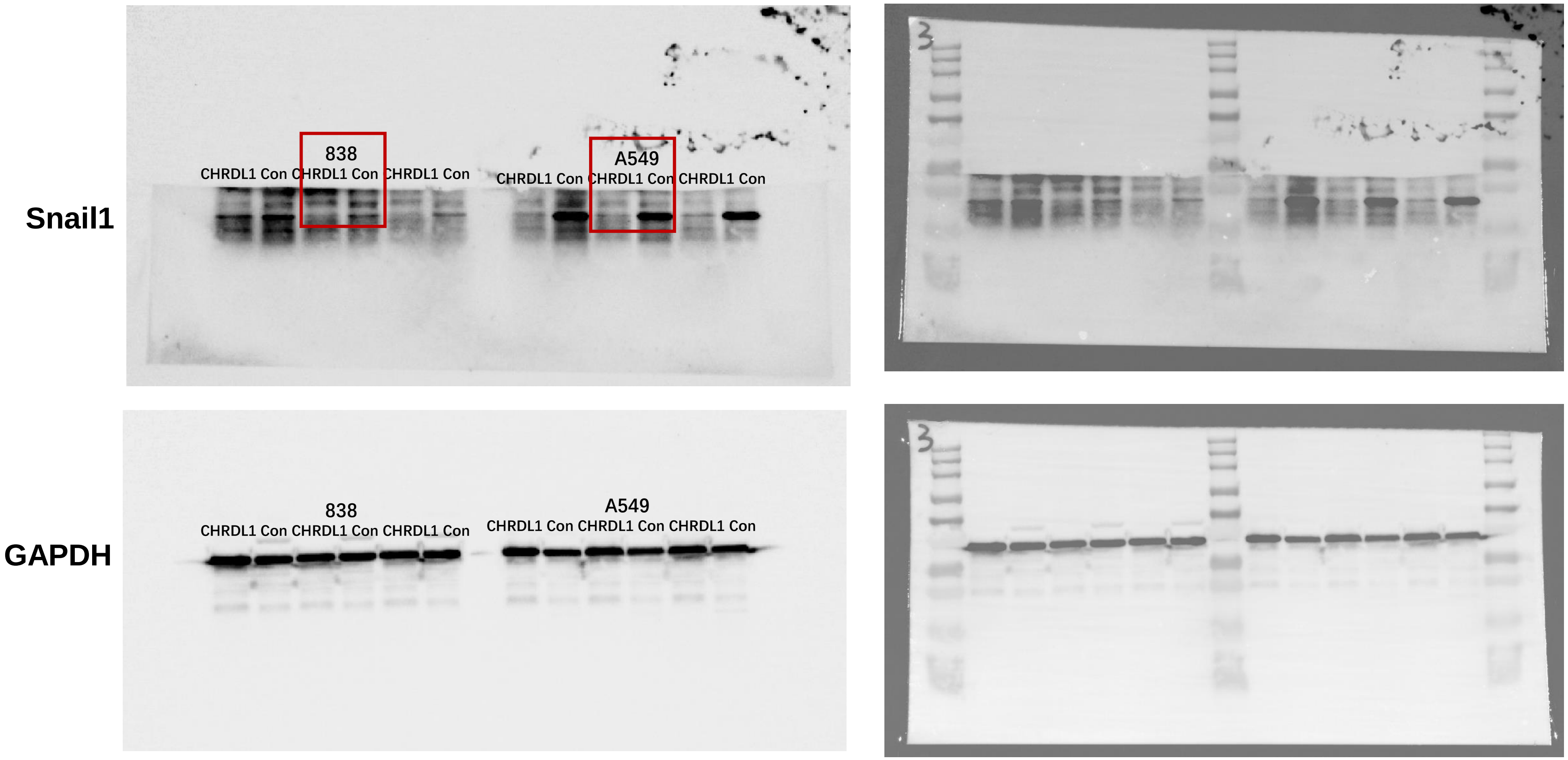
Snail1
838
CHRDL1 Con CHRDL1 Con CHRDL1 Con
A549
CHRDL1 Con CHRDL1 Con CHRDL1 Con
GAPDH
838
CHRDL1 Con CHRDL1 Con CHRDL1 Con
A549
CHRDL1 Con CHRDL1 Con CHRDL1 Con

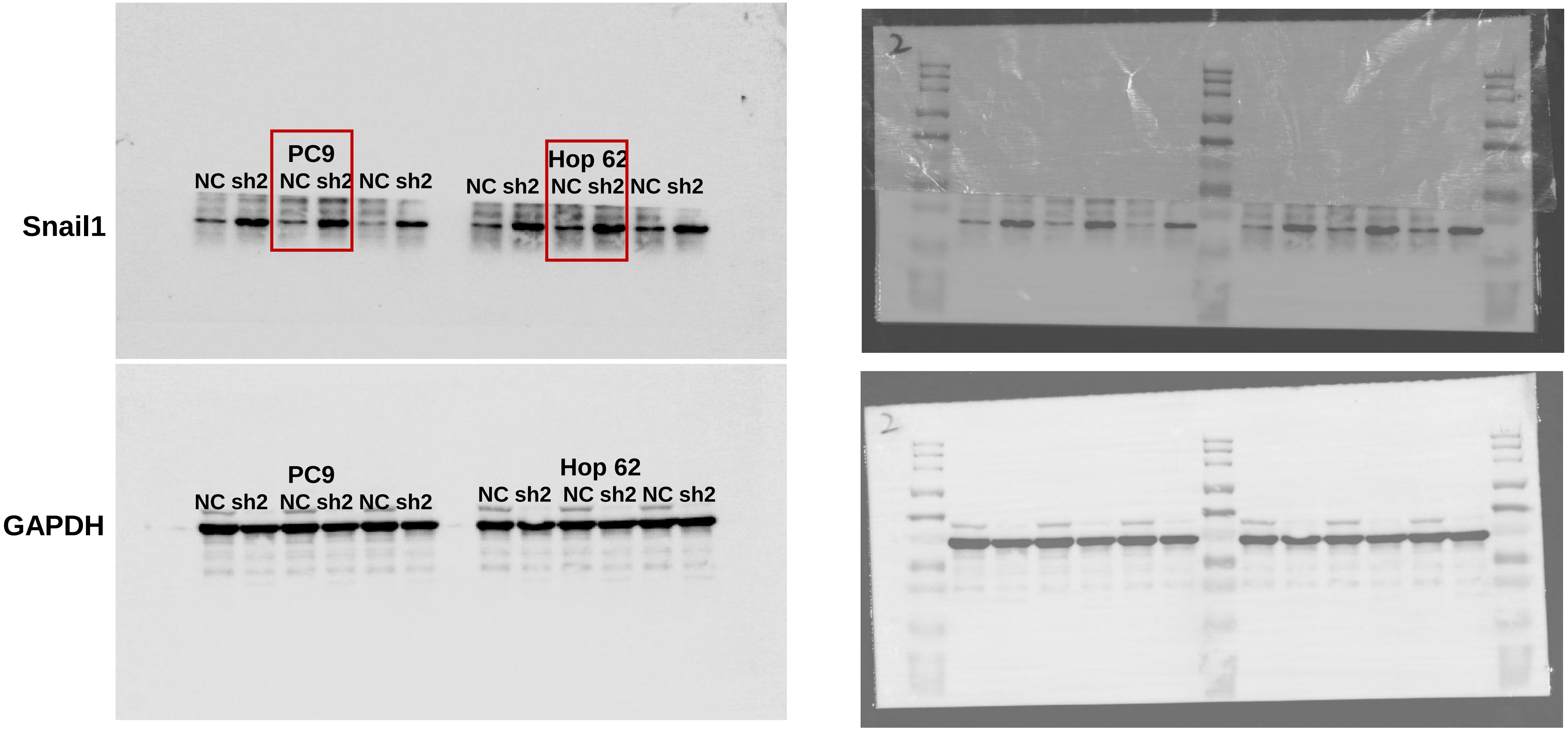
Snail1
PC9
NC sh2 NC sh2 NC sh2
Hop 62
NC sh2 NC sh2 NC sh2
GAPDH
PC9
NC sh2 NC sh2 NC sh2
Hop 62
NC sh2 NC sh2 NC sh2

Figure 10D

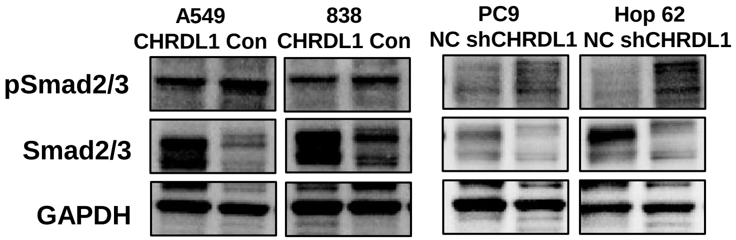

A549
CHRDL1 Con
838
CHRDL1 Con
PC9
NC shCHRDL1
Hop 62
NC shCHRDL1
pSmad2/3
Smad2/3
GAPDH

pSmad2/3

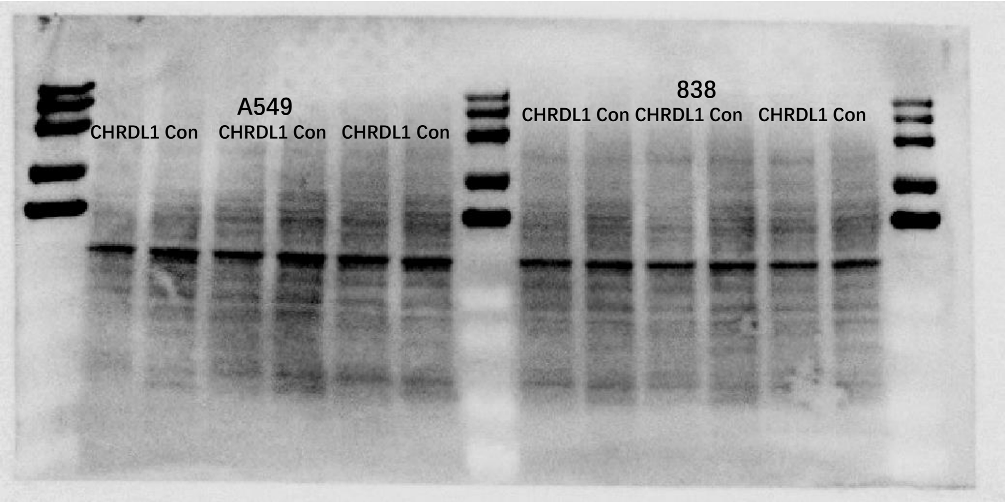

Smad2/3

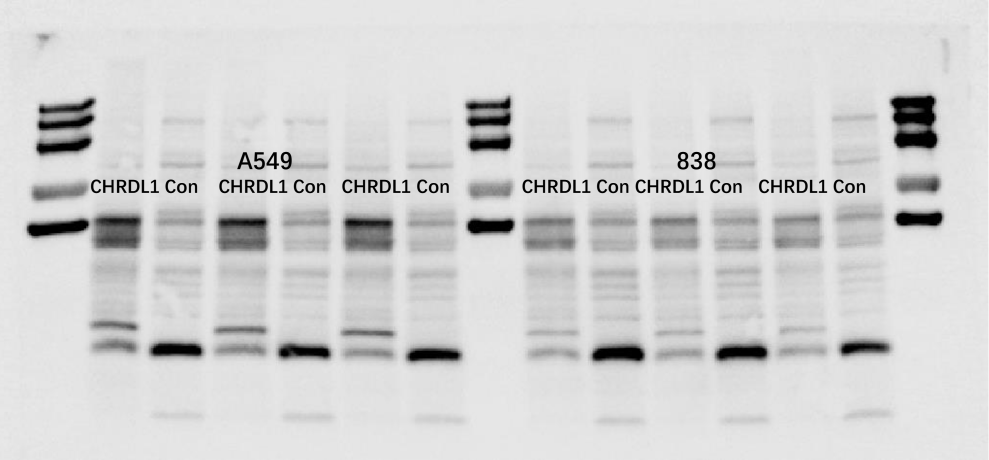

GAPDH

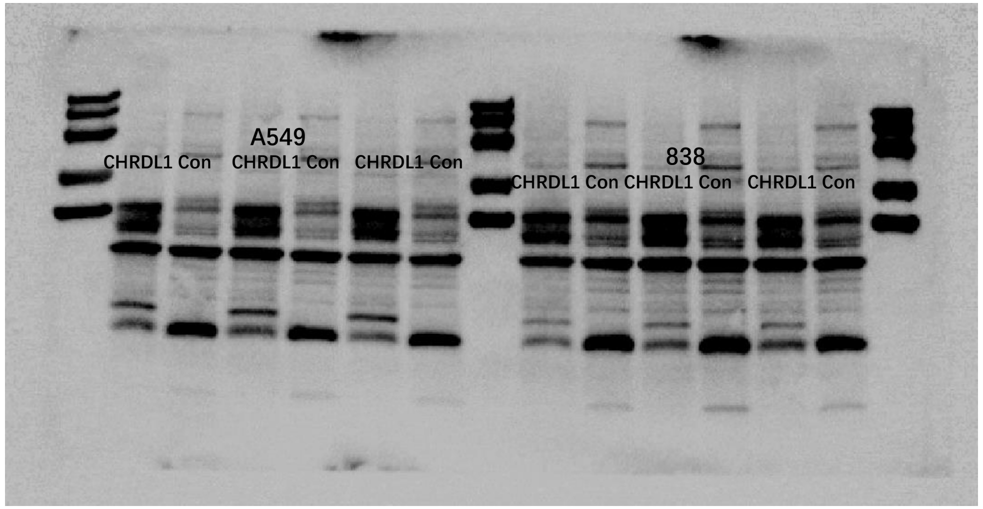

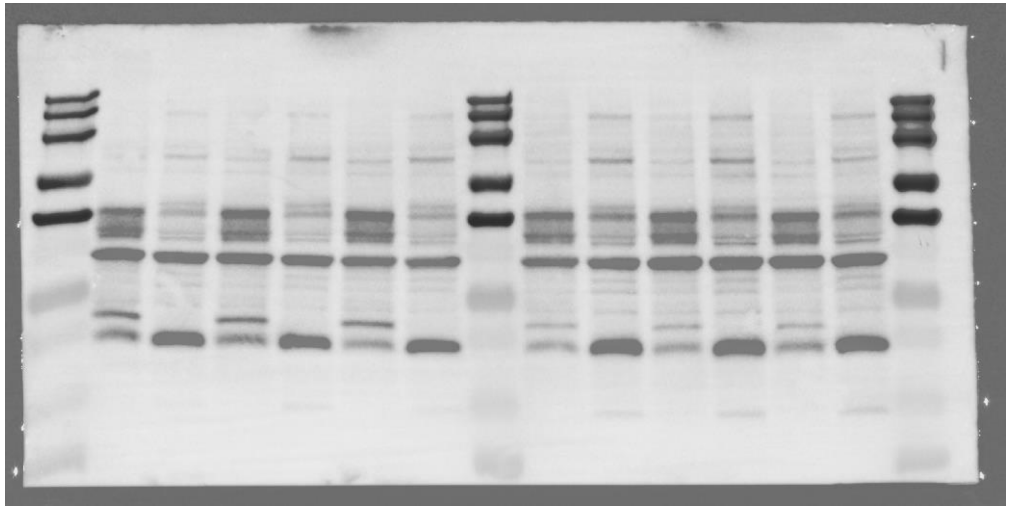

pSmad2/3

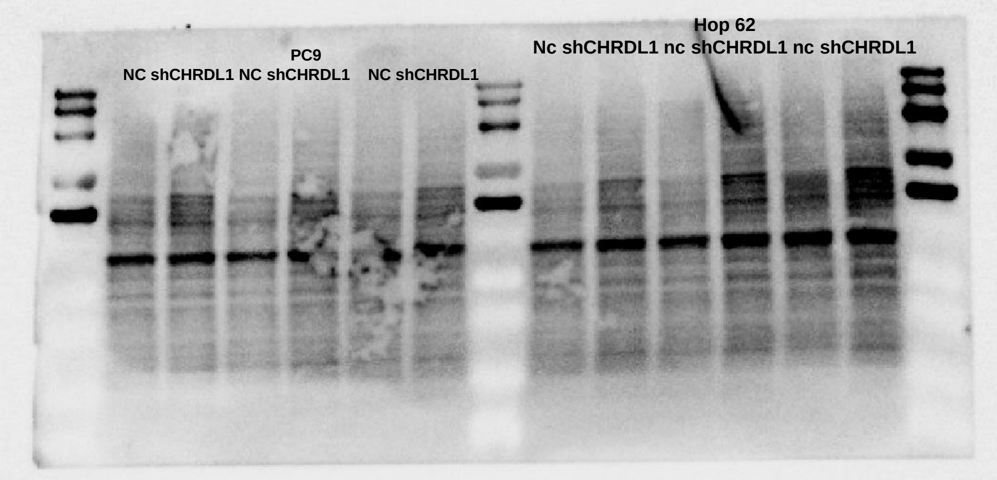

Smad2/3

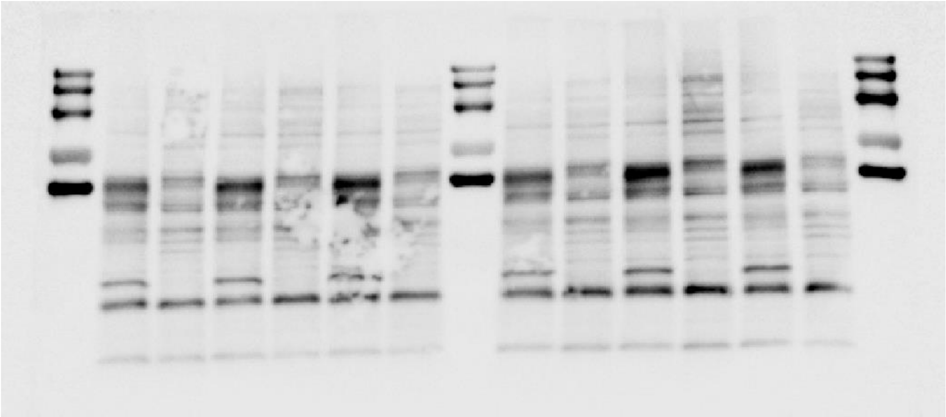

GAPDH

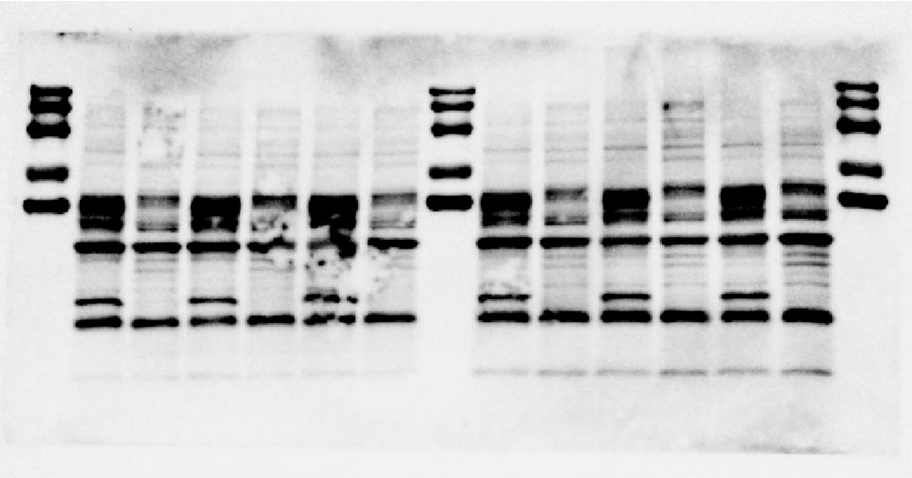

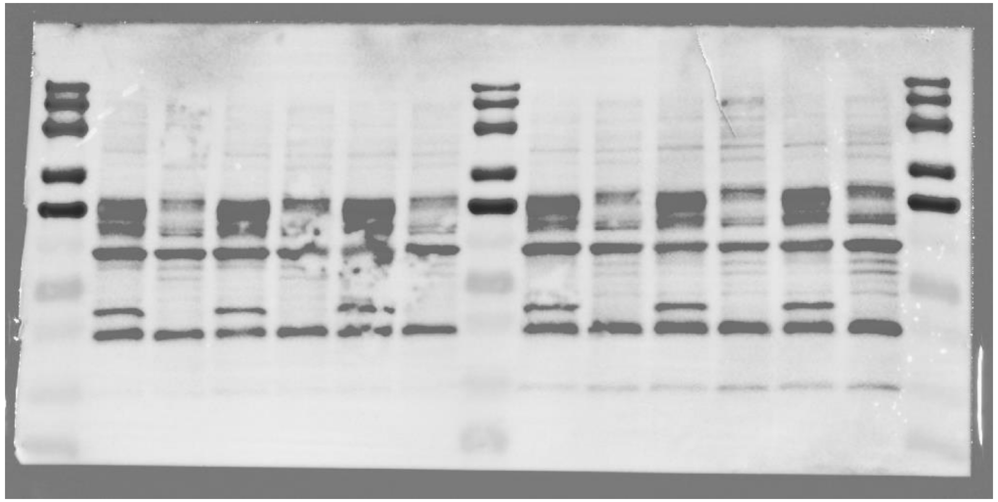

Supplement: Supplementary file 2 [file DataSheet2.zip › Supplementary figures and tables/WB figure Final.pdf]
